# RIF-YOLO-N: Lightweight P3 Residual Identity Fusion with Resolution-Guided Fine-Tuning for Tiny-Object Detection

**DOI:** 10.3390/jimaging12090450

**Published:** 2026-09-17

**Authors:** Mansoor Iqbal, Balaj Khalid, Syed Zarak Shah, Mishal Sarfraz, Khalid Munir, Zahid Ullah

**Affiliations:** 1 Big Earth Data Analytics Department, Eratosthenes Center of Excellence, Limassol 3012, Cyprus; 2 Viterbi School of Engineering, University of Southern California, Los Angeles, CA 90007, USA; 3 Department of Computational Sciences & Engineering, Harrisburg University of Science & Technology, Harrisburg, PA 17101, USA; 4 Department of Electrical and Computer Engineering, Oregon State University, Corvallis, OR 97331, USA; 5 Department of Computer Science, University of Alabama at Birmingham, Birmingham, AL 35294, USA; 6 Dipartimento di Elettronica, Informazione e Bioingegneria, Politecnico di Milano, 20133 Milano, Italy

**Keywords:** tiny-object detection, UAV imagery, RIF-YOLO-N, P3 feature enhancement, resolution-guided fine-tuning, lightweight object detection, tiny object detection

## Abstract

Tiny-object detection remains challenging because repeated downsampling degrades the limited spatial information available for small targets. This paper proposes RIF-YOLO-N, a lightweight detector that strengthens the finest P3 pathway of YOLOv8n through residual identity fusion (RIF). RIF combines neck-level semantic features with backbone-level spatial features via an identity-safe residual mechanism, preserving the original anchor-free P3/P4/P5 detection hierarchy. Resolution-guided fine-tuning (RGFT) further adapts the detector to higher-resolution inputs without adding a new detection head. The framework is evaluated on VisDrone, UAVDT, and VEDAI. At 1024×1024, RIF-YOLO-N with RGFT improves ${\mathrm{mAP}}_{50:95}$ from 0.235 to 0.243 on VisDrone, from 0.519 to 0.543 on UAVDT, and from 0.252 to 0.419 on VEDAI. On VisDrone, ${\mathrm{mAP}}_{50}$ also increases from 0.401 to 0.415, while model complexity rises only from 3.008M to 3.061M parameters and from 8.1 to 8.8 GFLOPs. Size-stratified analysis shows improvements for both tiny and small objects, with the larger ${\mathrm{AP}}_{50}$ gain observed for the small-object group. Ablation and resolution studies further support the selected P3 enhancement and show that RGFT performance depends on the chosen fine-tuning resolution. Overall, RIF-YOLO-N improves lightweight tiny-object detection without introducing an additional prediction scale or substantially redesigning the detector.

## 1. Introduction

Object detection is a fundamental computer vision task that supports localization and recognition in applications such as intelligent transportation, surveillance, robotics, remote sensing, and visual monitoring. Modern deep detectors have achieved strong performance by learning hierarchical feature representations from large-scale datasets [1,2,3]. However, detection accuracy decreases when scenes contain large-scale variation, dense object distributions, occlusion, motion blur, complex backgrounds, and objects that occupy only a small fraction of the image [4,5].

Tiny-object detection is a challenging problem due to the limited number of pixels on very small targets, weak texture, and poorly defined boundaries, which limit the discriminative information for classification and localization searching in tiny-object detection problems [6,7]. These objects can be suppressed further by repeated convolution and spatial downsampling layers, which will suppress the fine-grained information needed to represent them [8]. The problem applies to multiple visual domains, including traffic monitoring, surveillance, remote sensing, and UAV imagery. The UAV datasets are challenging since objects are often far away, occluded, and have low contrast with complex backgrounds. The preservation and enhancement of fine-scale features are important for the reliable detection of tiny objects because of these characteristics.

Recent studies address small- and tiny-object detection through several design strategies. Feature-pyramid enhancement and multi-scale feature fusion are widely used to preserve fine-scale information and strengthen cross-scale representations [9,10,11]. Related approaches improve multi-scale information interaction and feature aggregation for tiny targets [12]. Lightweight detector redesigns instead aim to improve small-object representation while controlling computational complexity [13,14,15]. Other YOLO-based methods combine lightweight architectures with specialized enhancement or fusion mechanisms for efficient UAV tiny-object detection [16,17,18]. High-resolution prediction branches can preserve spatial information but often increase computational cost, while attention and feature-pyramid redesigns may modify several detector components simultaneously. Such changes can make it difficult to isolate the contribution of a specific feature-enhancement mechanism. Lightweight detectors face the opposite problem: reducing model complexity can weaken the spatial or semantic information needed for tiny-object localization. This motivates a controlled feature-enhancement strategy that strengthens fine-scale representation without adding a new prediction scale or substantially redesigning the detector.

This paper proposes RIF-YOLO-N, a lightweight framework for tiny-object detection based on controlled enhancement of the existing YOLOv8n P3 pathway. The proposed residual identity fusion (RIF) module combines the semantically enriched neck-level P3 feature with the corresponding backbone-level spatial feature through an identity-safe residual mechanism. This design strengthens the finest existing detection pathway while preserving the original anchor-free P3/P4/P5 prediction hierarchy and the P4/P5 feature paths. Resolution-guided fine-tuning (RGFT) further adapts the enhanced representation to higher-resolution inputs without introducing an additional detection head. The resulting framework, therefore, targets improved tiny-object representation while maintaining a compact detector structure.

The proposed framework is evaluated on VisDrone, UAVDT, and VEDAI to examine its behavior under different tiny-object distributions and resolution conditions. The evaluation includes a controlled comparison with YOLOv8n, resolution sensitivity analysis, size-stratified and class-wise evaluation, detection diagnostics, component ablation, architecture selection experiments, computational analysis, and comparison with representative existing detectors. On VisDrone at 1024×1024, RIF-YOLO-N improves ${\mathrm{mAP}}_{50}$ from 0.401 to 0.415 and ${\mathrm{mAP}}_{50:95}$ from 0.235 to 0.243, while increasing the model size from 3.008M to 3.061M parameters and the computational cost from 8.1 to 8.8 GFLOPs. The detailed analyses show that the gains are concentrated mainly in tiny and small objects, while the cross-dataset results indicate that the effect of input resolution varies across datasets and evaluation metrics.

The main contributions of this paper are as follows:

- This study formulates lightweight tiny-object detection as a constrained fine-scale feature-representation problem, where the objective is to strengthen the spatially sensitive P3 representation while preserving the original P3/P4/P5 prediction hierarchy and limiting additional computational cost.
- This study introduces RIF-YOLO-N, which performs same-scale coupling of backbone- and neck-level P3 features through adaptive feature selection and identity-safe residual enhancement. The modification is confined to the existing P3 pathway and does not introduce an additional prediction scale or redesign the P4/P5 detection paths.
- This study incorporates resolution-guided fine-tuning as a training-only adaptation strategy that initializes higher-resolution optimization from the best base-resolution checkpoint while keeping the deployed detector architecture unchanged.
- The framework is evaluated on VisDrone, UAVDT, and VEDAI using controlled YOLOv8n comparisons, resolution-sensitivity analysis, size- and class-level evaluation, diagnostic assessment, component ablation, architecture selection, cross-dataset validation, and computational analysis to quantify both the benefits and limitations of the proposed design.

The remainder of this paper is organized as follows. Section 2 reviews recent studies on tiny-object detection, fine-scale feature preservation, multi-scale feature fusion, and lightweight detection architectures. Section 4 presents the RIF-YOLO-N architecture, the identity-safe RIF module, and the RGFT procedure. Section 5 describes the evaluation datasets, implementation settings, training protocol, and performance metrics. Section 6 presents the cross-dataset performance and comparative evaluation, fine-grained tiny-object analysis, detection diagnostics, ablation study, architecture-selection results, and discussion of the observed trends and limitations. Section 7 concludes the paper and outlines future research directions.

## 2. Related Work

### 2.1. Tiny- and Small-Object Detection

Tiny- and small-object detection remains difficult because small targets contain few pixels, weak texture, and poorly defined boundaries. Repeated downsampling in deep detectors can further suppress the spatial information required for accurate localization and classification. These limitations occur in surveillance, traffic monitoring, remote sensing, and UAV imagery, where targets may appear at long distances, overlap in dense scenes, or exhibit low contrast against complex backgrounds [4,5].

Recent methods improve tiny-object representation by strengthening fine-scale and multi-scale features. HS-FPN introduces high-frequency and spatial-perception components into the feature pyramid to preserve information useful for small targets [9]. FMFN-YOLO combines fine-grained feature preservation, multi-scale enhancement, and feature-pyramid balancing to improve small-object representation [19]. These studies demonstrate the importance of preserving spatial detail. However, they modify broader feature-pyramid or multi-scale processing structures, making it difficult to determine whether a targeted enhancement of an existing fine-scale detection feature is sufficient to improve tiny-object detection.

### 2.2. Feature Fusion and High-Resolution Preservation

Multi-scale feature fusion addresses scale variation by combining spatially detailed shallow features with semantically stronger deep features. For tiny objects, several studies further increase the contribution of high-resolution representations. SMA-YOLO introduces cross-scale fusion and an additional P2 detection head to improve small-object localization [10]. YOLO11s-UAV strengthens small-object features through content-aware feature reassembly and cross-scale interaction [20]. YOLO-BWS similarly introduces a higher-resolution detection head and bidirectional feature interaction for tiny-object detection [12]. Recent Transformer-based detectors have also addressed small-object representation without relying on the YOLO architecture. The authors of ref. [21] proposed FA-DETR, an end-to-end detector for UAV-based small-object detection that combines multi-scale edge enhancement, learnable positional embedding refinement, and multi-domain feature synthesis to strengthen boundary information, localization, and multi-scale feature representation in aerial imagery.

These approaches improve access to high-resolution information, but additional P2 prediction heads, high-resolution branches, and broader modifications to the feature pyramid increase structural and computational complexity. They also alter multiple detector pathways relative to the original architecture. Consequently, their performance gains cannot be attributed to a single controlled feature-enhancement operation. This leaves an important design question: whether fine-scale representation can be improved by reusing spatial information already available within the detector, without introducing another prediction scale.

### 2.3. Attention, Context Modeling, and Lightweight Detection

Attention and contextual modeling provide another strategy for improving weak object representations by emphasizing informative channels, spatial regions, boundaries, or surrounding context [22,23]. RTS-Net combines multi-scale feature fusion with coordinated attention and real-time feature extraction [24]. YOLO-MARS introduces a multi-level attention residual mechanism for small-target detection [25], while LRDS-YOLO incorporates resolution-aware hybrid attention within a lightweight feature-pyramid design [26]. These approaches can strengthen feature discrimination, but they introduce additional feature-processing mechanisms beyond the original detector pathway.

Lightweight detectors instead seek to reduce computational cost through efficient convolution, compact feature pyramids, simplified detection heads, and redundancy reduction [13,14,15,27]. Although these methods address deployment efficiency, many redesign several detector components simultaneously. This creates a trade-off between improving fine-scale representation and preserving a controlled lightweight architecture. A targeted modification that changes only the feature pathway most relevant to tiny objects would provide a clearer way to evaluate feature enhancement while limiting additional computation.

Table 1 summarizes the main design strategies represented by the reviewed methods and positions RIF-YOLO-N relative to these approaches.

### 2.4. Research Gap and Positioning

The literature shows that the remaining challenge is not the absence of feature-enhancement mechanisms, but the limited investigation of controlled fine-scale enhancement within an existing lightweight detection hierarchy. Repeated downsampling weakens the spatial cues required for tiny-object localization, while many existing methods compensate by introducing additional P2 heads, high-resolution branches, attention modules, or broader feature-pyramid redesigns. Although effective, such modifications often alter several detector components simultaneously, making it difficult to isolate whether targeted enhancement of the finest existing prediction pathway is sufficient.

The selection of P3 does not imply that P4 and P5 are unnecessary. P3 is targeted because its stride-8 representation provides the greatest spatial support among the existing prediction levels for tiny-object localization, whereas P4 and P5 are retained to provide complementary intermediate- and high-level semantic representations within the original detection hierarchy.

RIF-YOLO-N addresses this gap through a targeted modification of the existing YOLOv8n P3 pathway. P3 is selected because its stride-8 representation provides the strongest spatial support for tiny-object localization, while P4 and P5 are retained to preserve complementary intermediate- and high-level semantic information. The method performs same-scale coupling of backbone- and neck-level P3 features through adaptive gating and identity-safe residual enhancement, without requiring cross-scale resizing, an additional prediction scale, or changes to the anchor-free P3/P4/P5 detection hierarchy. RGFT further adapts the same architecture to higher-resolution inputs during training without introducing inference-time components. The contribution is therefore positioned as a controlled fine-scale feature-enhancement and training strategy rather than a broad detector redesign.

This distinction can also be expressed at the operator level. A generic cross-layer fusion mechanism can be abstracted as $F_{\mathrm{CL}}=\Phi \left(F_{l},R\left(F_{h}\right)\right)$, where R(·) performs spatial alignment when required and Φ(·) forms the fused representation. Similarly, a residual-attention mechanism can be represented as $F_{\mathrm{RA}}=X+\alpha A\left(X\right)⊙T\left(X\right)$, where attention reweights a transformed feature response. In contrast, RIF can be abstracted as $F_{3}^{\mathrm{rif}}=B_{3}+\gamma C\left(F_{3}^{n},F_{3}^{b}\right)$, with $\gamma_{0}=0$, where $B_{3}=S\left(F_{3}^{n}\right)$ denotes the preserved shortcut representation, S(·) denotes the shortcut mapping, and C(·) denotes the gated correction conditioned on the complementary backbone–neck P3 features. In the final channel-compatible RIF-YOLO-N configuration, S(·) is the identity mapping, so $B_{3}=F_{3}^{n}$. Thus, the term identity-safe refers primarily to the zero-initialized residual construction: the newly introduced correction branch contributes zero at initialization, while the original neck-level P3 representation is preserved exactly in the final configuration. Relative to conventional gated or residual feature-fusion mechanisms, RIF is distinguished by same-scale backbone–neck P3 coupling, selective introduction of complementary information through this identity-safe residual correction, and preservation of the original P3/P4/P5 prediction hierarchy. The novelty, therefore, lies in this controlled integration rather than in projection, gating, or residual learning individually. Table 2 summarizes these conceptual differences.

## 3. Problem Formulation

This study formulates lightweight tiny-object detection as a feature-representation and detector-design problem. The objective is to construct a lightweight detection framework that preserves sufficient spatial information for tiny targets while maintaining low computational complexity. RIF-YOLO-N is developed by redesigning the fine-scale detection pathway of YOLOv8n and inserting a residual identity fusion (RIF) module into the existing P3 pathway. The redesigned detector strengthens the representation used for tiny-object prediction while retaining the original P4/P5 pathways and anchor-free detection head. Let

(1) $$D={\left\{\left(I_{i},Y_{i}\right)\right\}}_{i=1}^{N}$$

denote an object-detection dataset, where $I_{i}\in R^{H_{i}\times W_{i}\times 3}$ represents an input image and $Y_{i}$ denotes its ground-truth annotation set. Each annotation set is defined as

(2) $$Y_{i}={\left\{\left(b_{ij},c_{ij}\right)\right\}}_{j=1}^{M_{i}},$$

where $M_{i}$ denotes the number of objects in image $I_{i}$, $c_{ij}\in \left\{1,\ldots ,K\right\}$ denotes the object class, and $b_{ij}$ represents the corresponding bounding box. Using normalized coordinates,

(3) $$b_{ij}=\left(x_{ij},y_{ij},w_{ij},h_{ij}\right),\;x_{ij},y_{ij},w_{ij},h_{ij}\in \left[0,1\right].$$

The central difficulty in tiny-object detection arises from the limited spatial support of very small targets. When an image is resized to an input resolution s×s, the approximate area of an object becomes

(4) $$A_{s}\left(b_{ij}\right)=w_{ij}h_{ij}s^{2}.$$

For a feature level *l* with stride $r_{l}$, the corresponding projected object area can be approximated as

(5) $$A_{l}\left(b_{ij};s\right)=\frac{A_{s}\left(b_{ij}\right)}{r_{l}^{2}}=\frac{w_{ij}h_{ij}s^{2}}{r_{l}^{2}}.$$

YOLOv8n performs prediction at three feature levels with strides

(6) $$r_{3}=8,\;r_{4}=16,\;r_{5}=32.$$

For the same object and input resolution,

(7) $$A_{3}\left(b_{ij};s\right)>A_{4}\left(b_{ij};s\right)>A_{5}\left(b_{ij};s\right),$$

Because the projected object area varies inversely with the squared feature stride, the spatial support across the three YOLOv8n prediction levels can be related directly as

$$A_{4}\left(b_{ij};s\right)=\frac{1}{4}A_{3}\left(b_{ij};s\right),\;A_{5}\left(b_{ij};s\right)=\frac{1}{16}A_{3}\left(b_{ij};s\right).$$

Thus, for the same object and input resolution, P3 retains four times the projected object area of P4 and sixteen times that of P5. At an input size of 1024×1024, the corresponding feature maps are 128×128, 64×64, and 32×32 for P3, P4, and P5, respectively. This higher spatial support makes P3 the most suitable existing level for targeted tiny-object enhancement. P4 and P5 are nevertheless retained because their progressively coarser representations provide complementary intermediate- and high-level semantic information, as summarized in Table 3. The design, therefore, enhances P3 for spatial detail rather than replacing the multi-scale P3/P4/P5 hierarchy.

The baseline YOLOv8n detector can be expressed as

(8) $$f_{\theta }^{\mathrm{base}}:I_{i}^{s}\to {\hat{Y}}_{i},$$

where $I_{i}^{s}$ is the resized input image, θ denotes the trainable parameters, and

(9) $${\hat{Y}}_{i}={\left\{\left({\hat{b}}_{ij},{\hat{c}}_{ij},{\hat{p}}_{ij}\right)\right\}}_{j=1}^{{\hat{M}}_{i}}$$

represents the predicted object set. Here, ${\hat{b}}_{ij}$ denotes the predicted bounding box, ${\hat{c}}_{ij}$ denotes the predicted class, ${\hat{p}}_{ij}$ denotes the confidence score, and ${\hat{M}}_{i}$ denotes the number of predicted objects.

The detector is trained by minimizing the standard detection objective

(10) $$\theta^{*}=\arg\min_{\theta }\frac{1}{N}\sum_{i=1}^{N}L\left(f_{\theta }\left(I_{i}^{s}\right),Y_{i}\right),$$

where the YOLOv8n loss combines localization, classification, and distribution-based bounding-box refinement:

(11) $$L=\lambda_{\mathrm{box}}L_{\mathrm{box}}+\lambda_{\mathrm{cls}}L_{\mathrm{cls}}+\lambda_{\mathrm{dfl}}L_{\mathrm{dfl}}.$$

RIF-YOLO-N redesigns the baseline detector by inserting a dedicated feature-enhancement transformation into the P3 pathway. Let the backbone generate

(12) $$\left\{F_{3}^{b},F_{4}^{b},F_{5}^{b}\right\}=B\left(I_{i}^{s}\right),$$

and let the neck produce

(13) $$\left\{F_{3}^{n},F_{4}^{n},F_{5}^{n}\right\}=N\left(F_{3}^{b},F_{4}^{b},F_{5}^{b}\right).$$

The proposed RIF transformation combines the backbone and neck representations at the same P3 scale:

(14) $$T_{\mathrm{rif}}:\left(F_{3}^{n},F_{3}^{b}\right)\to F_{3}^{\mathrm{rif}}.$$

The redesigned detector then performs prediction as

(15) $${\hat{Y}}_{i}^{\mathrm{rif}}=\mathrm{Detect}\left(F_{3}^{\mathrm{rif}},F_{4}^{n},F_{5}^{n}\right),$$

where $F_{3}^{\mathrm{rif}}$ replaces the original neck-level P3 feature, while $F_{4}^{n}$, $F_{5}^{n}$, and the anchor-free detection head remain unchanged. This redesign directly modifies the fine-scale representation used by the detector rather than introducing an additional P2 prediction scale or reconstructing the complete multi-scale hierarchy.

Because the framework targets lightweight tiny-object detection, the architectural redesign is also subject to complexity constraints. The detection head is preserved as

(16) $$H_{\mathrm{rif}}=H_{\mathrm{base}},$$

while the increases in parameter count and computational cost are bounded by

(17) $$\Delta P=P_{\mathrm{rif}}-P_{\mathrm{base}}\leq \epsilon_{P},$$

and

(18) $$\Delta F=F_{\mathrm{rif}}-F_{\mathrm{base}}\leq \epsilon_{F},$$

where P denotes the parameter count and F denotes the computational cost measured in  FLOPs.

The resulting optimization problem for the redesigned detector is therefore

(19) $$\begin{array}{cc} \theta_{\mathrm{rif}}^{*}=\; & \arg\min_{\theta }\frac{1}{N}\sum_{i=1}^{N}L\left(\mathrm{Detect}\left(T_{\mathrm{rif}}\left(F_{3}^{n},F_{3}^{b}\right),F_{4}^{n},F_{5}^{n}\right),Y_{i}\right)\\ s.t.\;\; & H_{\mathrm{rif}}=H_{\mathrm{base}},\\ \; & P_{\mathrm{rif}}-P_{\mathrm{base}}\leq \epsilon_{P},\\ \; & F_{\mathrm{rif}}-F_{\mathrm{base}}\leq \epsilon_{F}. \end{array}$$

This formulation defines RIF-YOLO-N as a lightweight detector framework specifically designed for tiny-object detection. The framework redesigns the fine-scale detection pathway by inserting the RIF module between the backbone and neck-level P3 representations, thereby strengthening spatially sensitive features before prediction. The P4/P5 pathways and anchor-free detection head are retained to control computational growth, while resolution-guided fine-tuning further adapts the redesigned detector to resolution-sensitive tiny-object patterns. Section 4 presents the RIF architecture and its training procedure in detail.

## 4. Methodology

### 4.1. Overview of the Proposed RIF-YOLO-N Framework

RIF-YOLO-N is a lightweight detection framework designed to improve tiny-object representation by redesigning the fine-scale pathway of YOLOv8n. The framework inserts a residual identity fusion (RIF) mechanism into the existing P3 pathway, where objects retain the largest spatial support among the standard P3/P4/P5 detection levels. The redesign strengthens the representation used for tiny-object prediction without introducing an additional detection scale or replacing the anchor-free detection head.

The framework follows three design principles. First, the framework targets the existing P3 pathway because its stride-8 representation retains four times the projected object area of P4 and sixteen times that of P5 for the same object, providing the strongest spatial support among the standard YOLOv8n prediction levels. P4 and P5 are retained unchanged to preserve their complementary intermediate- and high-level semantic information. Second, it couples the neck-level P3 representation with the corresponding backbone-level P3 representation at the same spatial scale, allowing semantic and spatial information to interact without cross-scale resizing. Third, it introduces the enhancement through a zero-initialized residual pathway so that the new branch contributes progressively during optimization. The P4/P5 architectural pathways, label assignment, bounding-box decoding, and post-processing operations retain their original YOLOv8n structure.

Let the backbone generate the multi-scale features

(20) $$\left\{F_{3}^{b},F_{4}^{b},F_{5}^{b}\right\}=B\left(I\right),$$

and let the neck produce

(21) $$\left\{F_{3}^{n},F_{4}^{n},F_{5}^{n}\right\}=N\left(F_{3}^{b},F_{4}^{b},F_{5}^{b}\right),$$

where B(·) and N(·) denote the backbone and neck, respectively. The baseline detector uses the resulting P3/P4/P5 hierarchy for prediction.

RIF-YOLO-N redesigns the P3 pathway through

(22) $$F_{3}^{\mathrm{rif}}=T_{\mathrm{rif}}\left(F_{3}^{n},F_{3}^{b}\right),$$

where $T_{\mathrm{rif}}\left(\cdot \right)$ denotes the proposed RIF transformation. The enhanced feature $F_{3}^{\mathrm{rif}}$ replaces the original P3 representation at the corresponding location in the detector. The subsequent bottom-up P3→P4→P5 aggregation structure and the three-scale Detect layer remain architecturally unchanged. Thus, the redesigned P3 representation can propagate through the existing downstream feature hierarchy without introducing a new prediction branch. As summarized theoretically in Section 2.4 and Table 2, RIF differs from generic cross-layer fusion by introducing the complementary same-scale P3 information as an identity-safe residual correction while retaining the existing prediction hierarchy, rather than constructing an additional cross-scale prediction pathway.

**Figure 1:**
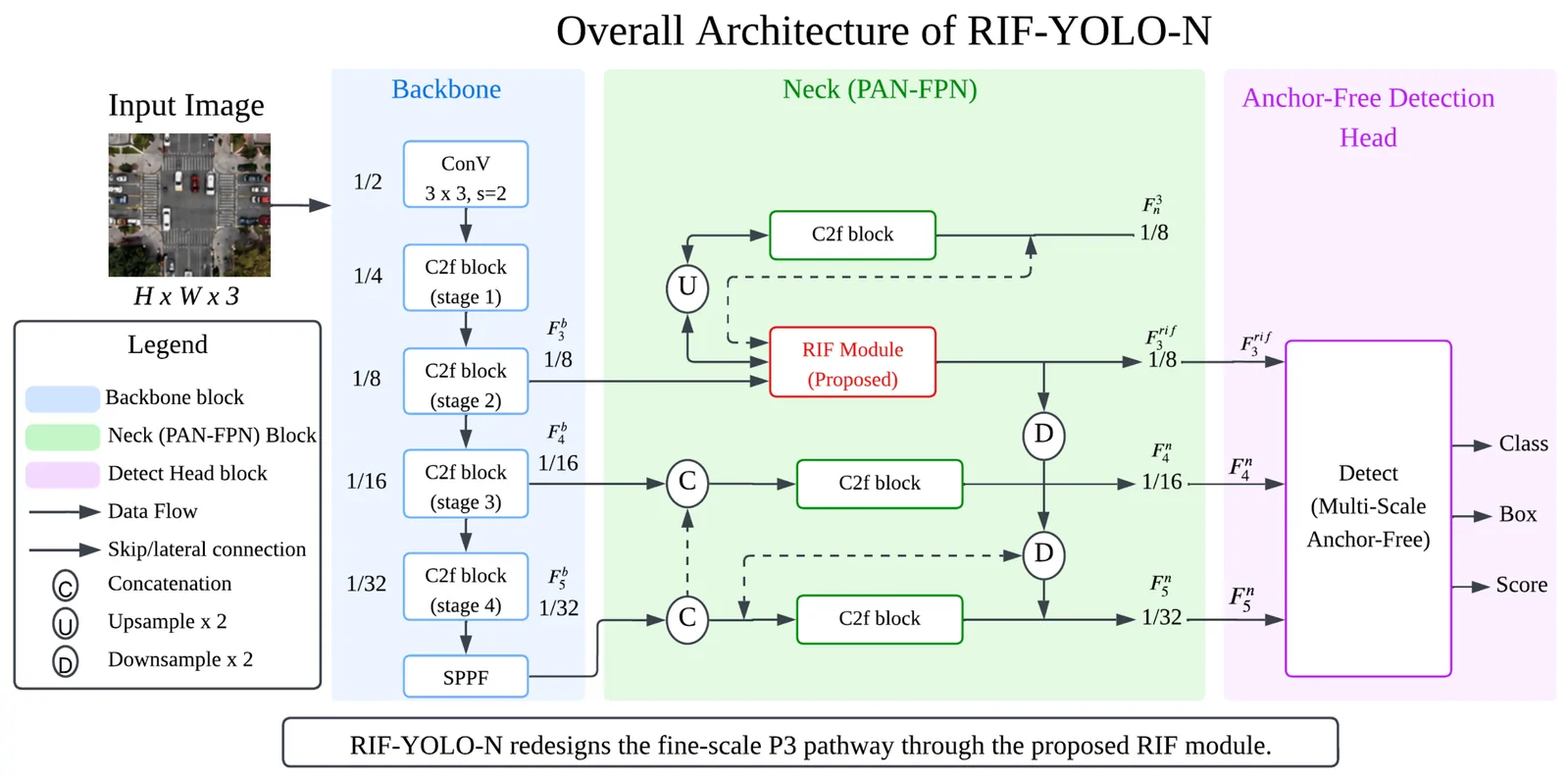
Overall architecture of RIF-YOLO-N. The proposed RIF module redesigns the fine-scale P3 pathway by fusing backbone and neck P3 features to generate $F_{3}^{\mathrm{rif}}$, while preserving the original P4/P5 pathways and multi-scale anchor-free detection head.

Figure 1 illustrates the overall RIF-YOLO-N architecture and the insertion of the proposed RIF module into the fine-scale P3 pathway.

### 4.2. Same-Scale Backbone–Neck P3 Feature Coupling

The RIF mechanism combines two complementary P3 representations. The neck-level feature $F_{3}^{n}$ contains semantically enriched information obtained through multi-scale aggregation, whereas the backbone-level feature $F_{3}^{b}$ retains fine spatial information before the full neck transformation. Their combination directly addresses the feature-representation problem defined in Section 3: tiny objects require spatially detailed features while still benefiting from semantic context.

The two features operate at stride 8 and therefore share the same spatial resolution:

(23) $$F_{3}^{n}\in R^{C_{n}\times H_{3}\times W_{3}},\;F_{3}^{b}\in R^{C_{b}\times H_{3}\times W_{3}},$$

where

(24) $$H_{3}=\frac{H}{8},\;W_{3}=\frac{W}{8}.$$

The RIF module separates the preserved main pathway from the projected features used to estimate the residual correction. The neck-level P3 feature first forms the shortcut representation

(25) $$B_{3}=S\left(F_{3}^{n}\right),$$

where S(·) is an identity mapping when the input and output channel dimensions match; otherwise, it performs channel alignment using a 1×1 convolution followed by batch normalization. In the final RIF-YOLO-N configuration, the P3 channel dimensions match and S(·), therefore reducing to the identity mapping. Independent of this shortcut, the neck- and backbone-level P3 features are projected into a common feature space for adaptive correction estimation:

(26) $$P_{n}=\phi_{n}\left(F_{3}^{n};\theta_{n}\right),$$

(27) $$P_{b}=\phi_{b}\left(F_{3}^{b};\theta_{b}\right),$$

where $\phi_{n}\left(\cdot \right)$ and $\phi_{b}\left(\cdot \right)$ denote 1×1 convolution, batch-normalization, and SiLU projection operations. The projected features satisfy $P_{n},P_{b}\in R^{C\times H_{3}\times W_{3}}$. These projections are used to estimate the adaptive residual correction and do not replace the preserved shortcut $B_{3}$. Because both selected P3 inputs operate at the same stride-8 resolution, no cross-scale resizing is required.

### 4.3. Adaptive Residual Feature Selection

Directly introducing all backbone responses into the enhanced P3 pathway can transmit redundant or weakly relevant information. RIF-YOLO-N therefore estimates an adaptive gate from the jointly projected backbone and neck representations:

(28) $$G=\sigma \;\left(\psi (\left[P_{n},P_{b}\right];\theta_{g})\right),$$

where [·,·] denotes channel-wise concatenation, ψ(·) is a 1×1 convolutional mapping, $\theta_{g}$ denotes its trainable parameters, and σ(·) denotes the sigmoid function. The resulting gate satisfies

(29) $$G\in {\left[0,1\right]}^{C\times H_{3}\times W_{3}},$$

and selectively modulates the projected backbone representation as

(30) $${\tilde{P}}_{b}=G⊙P_{b},$$

where ⊙ denotes element-wise multiplication. The neck projection $P_{n}$ conditions the gate together with $P_{b}$, whereas the gated backbone representation forms the input to the residual correction branch:

(31) $$C_{3}=\rho \left({\tilde{P}}_{b};\theta_{r}\right).$$

### 4.4. Identity-Safe Residual Enhancement

The gated complementary representation is refined using a standard 3×3 convolution followed by batch normalization and SiLU activation:

(32) $$\rho \left({\tilde{P}}_{b}\right)=\delta \;\left(\mathrm{BN}\;\left(W_{3\times 3}*{\tilde{P}}_{b}\right)\right),$$

where $W_{3\times 3}$ denotes a standard convolution and δ(·) denotes the SiLU activation. The final redesigned P3 representation is then

(33) $$F_{3}^{\mathrm{rif}}=B_{3}+\gamma C_{3},$$

where γ is a trainable residual scale initialized as

(34) $$\gamma_{0}=0.$$

At initialization, the residual correction is therefore inactive and

(35) $${\left.F_{3}^{\mathrm{rif}}\right|}_{\gamma =0}=B_{3}.$$

For the final RIF-YOLO-N configuration, the P3 channel dimensions match and the shortcut is an exact identity mapping, giving

(36) $$B_{3}=S\left(F_{3}^{n}\right)=F_{3}^{n},\;{\left.F_{3}^{\mathrm{rif}}\right|}_{\gamma =0}=F_{3}^{n}.$$

This relation defines the meaning of identity-safe. The term primarily refers to initialization stability: the newly inserted residual correction contributes exactly zero when γ=0, so the module initially reduces to its preserved shortcut pathway. In the final channel-compatible configuration, this shortcut is an exact identity mapping and therefore preserves the original neck-level P3 representation.

Combining the projection, gating, refinement, and residual operations gives the complete RIF transformation:

(37) $$F_{3}^{\mathrm{rif}}=S\left(F_{3}^{n}\right)+\gamma \;\rho \;\left(\sigma \;\left(\psi \;\left([\phi_{n}\left(F_{3}^{n}\right),\phi_{b}\left(F_{3}^{b}\right)]\right)\right)⊙\phi_{b}\left(F_{3}^{b}\right)\right),\;\gamma_{0}=0.$$

**Figure 2:**
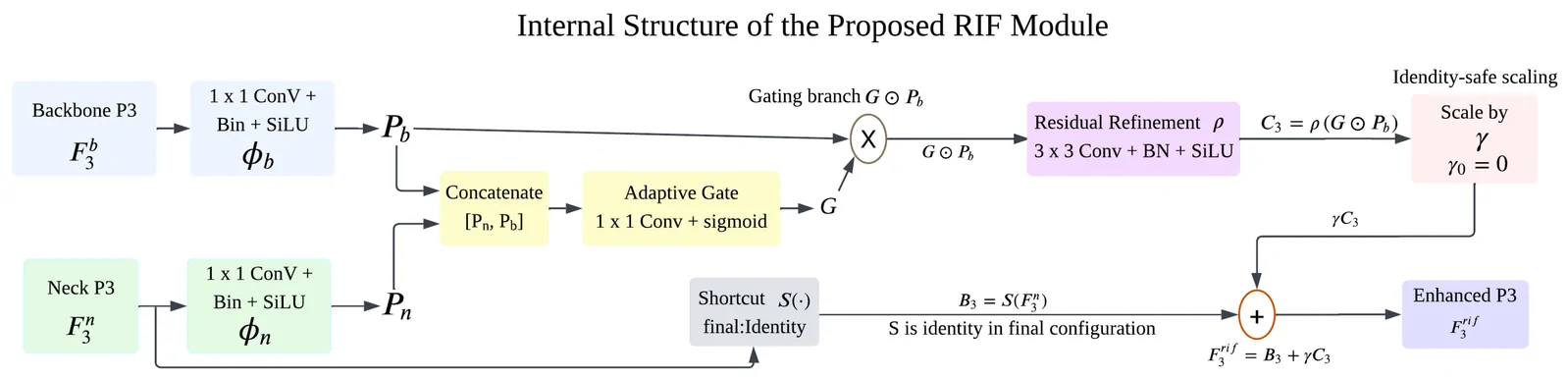
Internal structure of the proposed RIF module. Backbone and neck P3 features are projected into a common feature space, adaptively fused through a gating mechanism, and refined using an identity-safe residual branch to generate $F_{3}^{\mathrm{rif}}$.

Equation (37) provides the explicit realization of $T_{\mathrm{rif}}\left(F_{3}^{n},F_{3}^{b}\right)$ introduced in Section 3. The corresponding internal structure and information flow of the RIF module are illustrated in Figure 2.

### 4.5. Lightweight Architecture and Complexity Control

RIF-YOLO-N redesigns the fine-scale P3 pathway while controlling additional model complexity. The RIF module uses two 1×1 feature projections, a 1×1 gating operation, a standard 3×3 residual-refinement convolution, and a scalar residual scale. It does not introduce a P2 prediction head or an additional detection scale. For the final channel-compatible configuration, where the shortcut is parameter-free, and ignoring batch-normalization and bias terms, the additional parameter cost can be approximated as

(38) $$\Delta P_{\mathrm{rif}}\approx C\left(C_{n}+C_{b}\right)+2C^{2}+9C^{2}+1,$$

where $C_{n}$ and $C_{b}$ denote the neck- and backbone-input channel dimensions, *C* denotes the projected channel dimension, $C(C_{n}+C_{b})$ represents the two feature projections, $2C^{2}$ represents the gate mapping from 2C input channels to *C* outputs, $9C^{2}$ represents the standard 3×3 refinement convolution, and the final term corresponds to the residual scale γ. The refinement-convolution contribution is therefore

(39) $$P_{\rho }=9C^{2}.$$

The architectural design follows the constraints established in Section 3:

(40) $$H_{\mathrm{rif}}=H_{\mathrm{base}},\;\Delta P\leq \epsilon_{P},\;\Delta F\leq \epsilon_{F},$$

The first condition preserves the original anchor-free Detect operator, while the remaining conditions constrain parameter and computational growth. The resulting framework, therefore, constitutes a targeted P3-pathway redesign rather than an additional high-resolution detection architecture.

### 4.6. Resolution-Guided Fine-Tuning

The architectural redesign is complemented by resolution-guided fine-tuning (RGFT). RGFT uses a two-stage optimization process to expose the redesigned detector to both base-resolution and higher-resolution tiny-object representations while keeping the network architecture unchanged between stages.

In the first stage, RIF-YOLO-N is trained at a base resolution $s_{b}$:

(41) $$\theta_{b}^{*}=\arg\min_{\theta }\frac{1}{N}\sum_{i=1}^{N}L\left(f_{\theta }\left(I_{i}^{s_{b}}\right),Y_{i}\right).$$

The training objective retains the original YOLOv8n detection loss:

(42) $$L=\lambda_{\mathrm{box}}L_{\mathrm{box}}+\lambda_{\mathrm{cls}}L_{\mathrm{cls}}+\lambda_{\mathrm{dfl}}L_{\mathrm{dfl}},$$

where $L_{\mathrm{box}}$, $L_{\mathrm{cls}}$, and $L_{\mathrm{dfl}}$ denote localization, classification, and distribution focal losses, respectively.

In the second stage, the best base-stage checkpoint initializes training at a higher resolution $s_{h}$:

(43) $$\theta_{h}^{*}=\arg\min_{\theta }\frac{1}{N}\sum_{i=1}^{N}L\left(f_{\theta }\left(I_{i}^{s_{h}}\right),Y_{i}\right),\;\theta \leftarrow \theta_{b}^{*},\;s_{h}>s_{b}.$$

For a normalized bounding box $b_{j}=\left(x_{j},y_{j},w_{j},h_{j}\right)$, its approximate pixel area at resolution *s* is

(44) $$A_{s}\left(b_{j}\right)=w_{j}h_{j}s^{2}.$$

Increasing the input resolution from $s_{b}$ to $s_{h}$, therefore, changes the available object area according to

(45) $$\frac{A_{s_{h}}\left(b_{j}\right)}{A_{s_{b}}\left(b_{j}\right)}={\left(\frac{s_{h}}{s_{b}}\right)}^{2}.$$

The target resolution $s_{h}$ is treated as a training hyperparameter rather than a fixed property of RGFT. For VisDrone and UAVDT, 832×832 was empirically selected during preliminary development as a practical intermediate high-resolution setting, rather than through an exhaustive resolution search. Relative to the base resolution $s_{b}=640$, Equation (45) gives

$${\left(\frac{832}{640}\right)}^{2}\approx 1.69,$$

indicating that a normalized object occupies approximately 69% more pixel area at 832×832 than at 640×640. This increase provides greater spatial support for tiny targets while avoiding the larger memory and training cost associated with directly performing all high-resolution fine-tuning at 1024×1024. Therefore, 832×832 is used as an empirically selected operating point for VisDrone and UAVDT and is not claimed to be a theoretically or universally optimal RGFT resolution. The experiments on VEDAI further examine this dataset dependence using separate resolution-matched fine-tuning configurations.

RGFT does not introduce an additional architectural component at inference. Instead, it adapts the redesigned detector’s parameters to the larger spatial representation available at higher input resolutions. RIF and RGFT therefore have complementary roles: RIF modifies the P3 feature pathway and introduces the architectural overhead, whereas RGFT is a training-only strategy that adapts the same detector to higher-resolution inputs without adding inference-time components.

**Figure 3:**
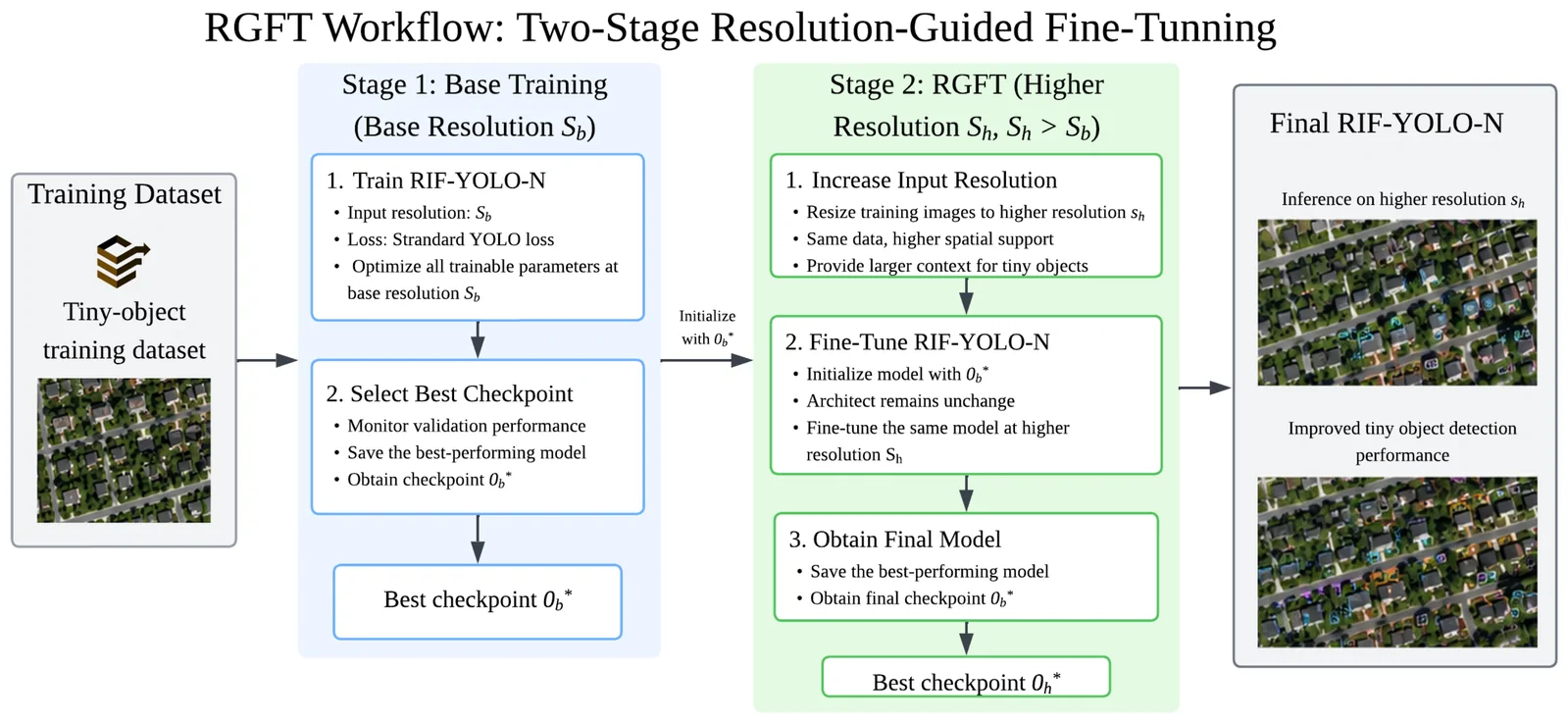
RGFT workflow for RIF-YOLO-N. The detector is first trained at base resolution $s_{b}$ to obtain the best checkpoint $\theta_{b}^{*}$, and is then fine-tuned at a higher resolution $s_{h}$ using the same architecture to produce the final checkpoint $\theta_{h}^{*}$.

Figure 3 summarizes the two-stage RGFT procedure, in which RIF-YOLO-N is first trained at the base resolution $s_{b}$ and then fine-tuned at a higher resolution $s_{h}$ using the best base-stage checkpoint while preserving the same architecture.

Algorithm 1 summarizes the complete training and inference procedure.
**Algorithm 1** RIF-YOLO-N training and inference procedure.**Require:** Training dataset D, base resolution $s_{b}$, fine-tuning resolution $s_{h}$, and YOLOv8n initialization**Ensure:** Trained RIF-YOLO-N detector $f_{\theta_{h}^{*}}$  1:Initialize the YOLOv8n backbone, neck, and anchor-free Detect layer  2:Insert the RIF transformation into the P3 pathway  3:Initialize the residual scale $\gamma_{0}=0$  4:**for** each base-stage training iteration at resolution $s_{b}$ **do**  5:     Extract backbone features $\left\{F_{3}^{b},F_{4}^{b},F_{5}^{b}\right\}$  6:     Generate neck features including $F_{3}^{n}$  7:     Form the preserved shortcut $B_{3}=S\left(F_{3}^{n}\right)$  8:     Compute projected features $P_{n}=\phi_{n}\left(F_{3}^{n}\right)$ and $P_{b}=\phi_{b}\left(F_{3}^{b}\right)$  9:     Compute the adaptive gate $G=\sigma (\psi \left(\left[P_{n},P_{b}\right]\right))$10:     Compute the gated backbone feature ${\tilde{P}}_{b}=G⊙P_{b}$11:     Compute the residual correction $C_{3}=\rho \left({\tilde{P}}_{b}\right)$12:     Compute the enhanced P3 feature $F_{3}^{\mathrm{rif}}=B_{3}+\gamma C_{3}$13:     Propagate $F_{3}^{\mathrm{rif}}$ through the existing downstream P3/P4/P5 hierarchy14:     Predict objects using the unchanged anchor-free Detect layer15:     Update the trainable parameters using $L_{\mathrm{box}}$, $L_{\mathrm{cls}}$, and $L_{\mathrm{dfl}}$16:**end for**17:Save the best base-stage checkpoint $\theta_{b}^{*}$18:Initialize the RGFT stage with $\theta_{b}^{*}$19:Fine-tune the same architecture at resolution $s_{h}$ to obtain $\theta_{h}^{*}$20:During inference, use the trained P3/P4/P5 detector and standard post-processing

## 5. Performance Validation

This section describes the validation design used to evaluate RIF-YOLO-N. The evaluation focuses on detection accuracy, localization quality, computational efficiency, and cross-dataset behavior. Three aerial object detection datasets are used to assess the proposed model under different scene conditions: dense drone-based traffic scenes, vehicle-focused UAV imagery, and tiny-object aerial imagery. All experiments use controlled training, validation, and reporting protocols to support fair comparison between YOLOv8n and RIF-YOLO-N.

### 5.1. Datasets and Visual Characteristics

The performance evaluation uses VisDrone [28], UAVDT [29], and VEDAI [30] to assess the proposed method under complementary conditions for tiny-object detection. VisDrone contains dense multi-class scenes with substantial scale variation, occlusion, and background clutter. UAVDT provides vehicle-focused UAV imagery with fewer categories and strong class imbalance. VEDAI contains overhead aerial imagery of multiple vehicle types and provides a distinct setting for evaluating small-object localization at high spatial resolution. Table 4 summarizes the main properties of the datasets used in this study.

For VisDrone, the validation set contains 38,759 annotated objects and is strongly dominated by tiny and small instances. In the converted validation set, 25,967 objects are categorized as tiny and 11,894 as small, together accounting for 97.69% of all annotations. This distribution provides a demanding setting for evaluating whether the proposed P3 feature enhancement improves feature representation under dense tiny-object conditions. This study uses the official VisDrone training and validation splits and does not use a separate, independently annotated VisDrone test split. Therefore, checkpoint selection and the reported VisDrone resolution, class-wise, size-stratified, ablation, architecture-selection, and aggregate performance analyses are validation-based evaluations and should not be interpreted as independent test-set estimates.

UAVDT is used as an external vehicle-focused benchmark. The local YOLO-format split contains 1266 training images, 271 validation images, and 272 test images, with three classes: car, truck, and bus. The annotation distribution is strongly imbalanced toward the car class. This setting evaluates whether RIF-YOLO-N remains effective when the number of categories is limited and the baseline detector already provides strong detection performance.

VEDAI provides an additional high-resolution aerial benchmark for evaluating small vehicle instances. The prepared YOLO-format dataset contains 968 training images, 121 validation images, and 121 test images. Only the visible-light images are used. The dataset contains nine classes: car, truck, tractor, camping-car, van, other, pickup, boat, and plane. The validation split contains 368 retained object instances, while the held-out test split contains 365 instances. The original oriented annotations are converted to axis-aligned bounding boxes to match the detection formulation used by RIF-YOLO-N. This dataset, therefore, provides a complementary setting for evaluating resolution-sensitive tiny-object detection and the effect of RGFT at high input resolutions.

**Figure 4:**
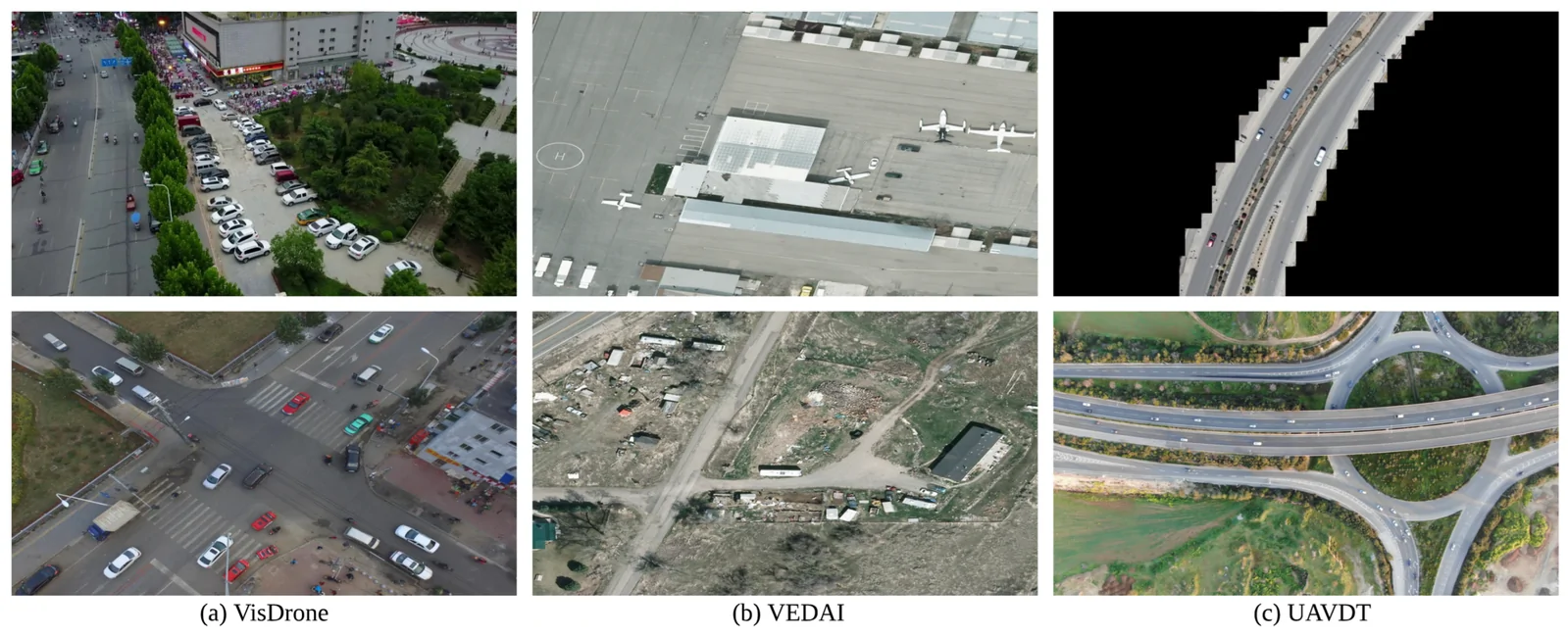
Representative sample images from the three evaluation datasets: (**a**) VisDrone, (**b**) VEDAI, and (**c**) UAVDT, illustrating variations in scene density, object scale, viewpoint, and background complexity.

Representative examples from the three datasets are shown in Figure 4. The visual samples illustrate differences in object density, viewpoint, object scale, and scene complexity across the datasets.

### 5.2. Experimental Environment and Reproducibility Configuration

All experiments were executed on the same workstation to reduce hardware-related variation in training and inference measurements. Table 5 reports the hardware and software environment used for model training, validation, and result analysis.

Table 6 summarizes the main reproducibility settings. The baseline YOLOv8n and RIF-YOLO-N models are trained under matched settings for each dataset. The same dataset split, input resolution, validation protocol, and metric computation are used when comparing the baseline and proposed model.

The implementation includes model configuration files, dataset configuration files, custom module registration, training scripts, validation scripts, and result-generation utilities. The VisDrone, UAVDT, and VEDAI datasets are not redistributed with the source code. Users must download the datasets from their official sources and use the provided conversion or configuration scripts to reproduce the experiments.

### 5.3. Evaluation Metrics

The evaluation uses both detection metrics and computational metrics. This combination is necessary because the goal of RIF-YOLO-N is not only to improve detection accuracy, but also to preserve the lightweight behavior of YOLOv8n.

For a predicted bounding box $\hat{b}$ and a ground-truth bounding box *b*, the intersection-over-union is defined as

(46) $$\mathrm{IoU}\left(\hat{b},b\right)=\frac{|\hat{b}\cap b|}{|\hat{b}\cup b|}.$$

A prediction is counted as a true positive when its IoU with a ground-truth box is greater than or equal to the selected threshold and the predicted class is correct. False positives represent incorrect or duplicate predictions, while false negatives represent missed ground-truth objects.

Precision measures the reliability of predicted detections:

(47) $$\mathrm{Precision}=\frac{TP}{TP+FP}.$$

Recall measures the model’s ability to recover ground-truth objects:

(48) $$\mathrm{Recall}=\frac{TP}{TP+FN}.$$

Recall is especially important in tiny-object detection because missed objects are common when objects occupy very small regions in the image.

Average precision for class *k* at IoU threshold τ is computed from the precision–recall curve:

(49) $${\mathrm{AP}}_{k,\tau }=\int_{0}^{1}P_{k,\tau }\left(r\right)\;dr,$$

where $P_{k,\tau }\left(r\right)$ denotes precision as a function of recall *r*. The mean average precision at IoU threshold τ is

(50) $${\mathrm{mAP}}_{\tau }=\frac{1}{K}\sum_{k=1}^{K}{\mathrm{AP}}_{k,\tau },$$

where *K* is the number of object classes.

The metric ${\mathrm{mAP}}_{50}$ uses τ=0.50:

(51) $${\mathrm{mAP}}_{50}={\mathrm{mAP}}_{\tau =0.50}.$$

This metric reflects general object detection ability. The stricter ${\mathrm{mAP}}_{50:95}$ metric averages mAP across ten IoU thresholds from 0.50 to 0.95:

(52) $${\mathrm{mAP}}_{50:95}=\frac{1}{10}\sum_{\tau \in \{0.50,0.55,\ldots ,0.95\}}{\mathrm{mAP}}_{\tau }.$$

This metric gives a stricter assessment of localization quality and is important for evaluating whether the predicted boxes align well with small aerial objects.

The computational metrics include parameter count, GFLOPs, and inference time. Parameter count measures model size:

(53) $$P=\sum_{l=1}^{L}\left|\theta_{l}\right|,$$

where $\theta_{l}$ denotes the learnable parameters in layer *l*. GFLOPs estimate the number of floating-point operations needed for one forward pass:

(54) $$F_{G}=\frac{F}{10^{9}}.$$

Forward-pass latency is measured using a controlled GPU timing protocol rather than by averaging the complete validation pipeline. After $N_{w}$ warm-up iterations, the mean forward-pass latency over $N_{t}$ timed iterations is computed as

(55) $${\bar{t}}_{\mathrm{fwd}}=\frac{1}{N_{t}}\sum_{i=1}^{N_{t}}t_{i},$$

where $t_{i}$ denotes the synchronized latency of the *i*-th model forward pass. In the reported runtime benchmark, $N_{w}=50$ warm-up iterations and $N_{t}=200$ timed iterations are used at 1024×1024 with batch size one. The reported value is the mean ± standard deviation across these 200 timed passes. The benchmark measures model forward computation and does not represent end-to-end application latency including data loading, pre-processing, post-processing, or visualization.

### 5.4. Simulation and Result-Generation Protocol

The result-generation process follows a controlled protocol. First, each dataset is prepared independently and converted to the YOLO normalized annotation format. The dataset configuration files define the training, validation, and test image paths, number of classes, and class names. A sanity validation step is performed after conversion to confirm that images and labels are readable by the Ultralytics framework.

Second, YOLOv8n is trained as the baseline detector for each dataset. RIF-YOLO-N is then trained under the same dataset split and matched training settings. The proposed model uses the same anchor-free detection head as YOLOv8n, while the RIF module modifies only the P3 feature pathway.

Third, the best checkpoint from each training run is selected according to validation performance. The selected checkpoint is evaluated at the target validation resolutions. For the full RIF-YOLO-N pipeline, resolution-guided fine-tuning is applied from the best base-stage checkpoint, and the fine-tuned model is evaluated again under the same metric set.

Third, the best checkpoint from each training run is selected according to validation performance. For VisDrone and UAVDT, the reported quantitative analyses are subsequently performed on their respective validation splits; therefore, these results represent validation-based evaluation rather than independent test-set performance. For VEDAI, the validation split is used for checkpoint selection, whereas the final reported detector performance is evaluated on the separate held-out test split. To separate the effect of RGFT from the proposed architecture, an additional YOLOv8n control was fine-tuned from its best 640×640 checkpoint at 832×832 using the same RGFT procedure as RIF-YOLO-N and was subsequently evaluated at 1024×1024. This provides a training-strategy-matched comparison between YOLOv8n and RIF-YOLO-N. For VisDrone and UAVDT, the RGFT stage uses 832×832 as the empirically selected high-resolution fine-tuning target. The resulting checkpoints are subsequently evaluated at several input resolutions to examine resolution sensitivity. For VEDAI, separate RGFT checkpoints are fine-tuned and evaluated at 832×832 and 1024×1024, allowing the effect of resolution-matched adaptation to be examined explicitly. Thus, the RGFT target resolution is treated as an experimental hyperparameter rather than a fixed architectural setting.

Fourth, component-level ablation is conducted on RIF-YOLO-N by removing the adaptive gate and the identity-safe residual scale. These ablation variants are trained and evaluated under the same VisDrone protocol to isolate the contribution of each component. The final results report accuracy metrics, class-wise behavior, computational cost, and inference time.

This protocol separates three forms of evidence: baseline comparison, component-level ablation, and external dataset validation. This separation allows the results to show whether RIF-YOLO-N improves over YOLOv8n, whether each component contributes to the final model, and whether the method generalizes beyond a single dataset.

## 6. Results and Discussion

This section presents a systematic performance analysis of RIF-YOLO-N across primary benchmark evaluation, object-scale behavior, class-level detection, diagnostic visualization, external validation, and component-level contribution. The analysis focuses on two main questions: whether the proposed framework improves tiny-object detection while preserving lightweight complexity, and whether the observed gains remain consistent across different aerial detection settings. The evaluation therefore reports detection accuracy, localization quality, computational cost, resolution sensitivity, class-wise AP behavior, object-size-specific AP, confusion patterns, qualitative detections, and ablation results.

On the VisDrone validation set at 1024×1024, RIF-YOLO-N improves ${\mathrm{mAP}}_{50}$ from 0.401 to 0.415 and ${\mathrm{mAP}}_{50:95}$ from 0.235 to 0.243, while precision and recall increase from 0.507 and 0.411 to 0.520 and 0.424, respectively. The ${\mathrm{mAP}}_{50:95}$ improvement is modest in absolute magnitude and is therefore interpreted together with the computational and fine-grained evidence rather than as a large aggregate gain. The model adds only 0.053M parameters and 0.7 GFLOPs, while the size-stratified analysis shows ${\mathrm{AP}}_{50}$ improvements from 0.1298 to 0.1346 for tiny objects and from 0.3771 to 0.3852 for small objects. Class-wise evaluation further shows positive ${\mathrm{AP}}_{50}$ changes in seven of ten VisDrone categories, although visually ambiguous categories remain challenging. The external datasets exhibit different response magnitudes: at 1024×1024, ${\mathrm{mAP}}_{50:95}$ increases by 0.024 on UAVDT and by 0.167 on VEDAI. These non-uniform results indicate that the benefit of the proposed framework depends on object scale, class composition, scene characteristics, and operating resolution. The following analyses therefore examine not only aggregate accuracy, but also resolution sensitivity, object-size behavior, class-level errors, diagnostic outputs, component contributions, and computational cost to identify where the method is effective and where its limitations remain.

### 6.1. Performance of the Proposed RIF-YOLO-N Model

The performance of the proposed RIF-YOLO-N model with RGFT was evaluated on VisDrone, UAVDT, and VEDAI. To provide a consistent cross-dataset assessment, Table 7 reports the final results at the common input resolution of 1024×1024.

RIF-YOLO-N achieves ${\mathrm{mAP}}_{50}$ values of 0.415, 0.862, and 0.669 on VisDrone, UAVDT, and VEDAI, respectively. The corresponding ${\mathrm{mAP}}_{50:95}$ values are 0.243, 0.543, and 0.419. The model achieves its highest overall detection performance on UAVDT, while the VEDAI results also show strong detection and localization performance in a different aerial scene with a different object distribution. VisDrone remains the more challenging evaluation setting, consistent with its dense scenes and high proportion of tiny and small objects.

#### 6.1.1. Robustness to Random Initialization

To assess the sensitivity of the proposed framework to random initialization, the complete RIF-YOLO-N training procedure was repeated using three independent seeds, {5,42,123}, on VisDrone, UAVDT, and VEDAI. Table 8 reports the mean ± sample standard deviation across the three runs. VisDrone and UAVDT show low run-to-run variation, with ${\mathrm{mAP}}_{50:95}$ standard deviations of 0.0023 and 0.0012, respectively. VEDAI exhibits greater variability, with a ${\mathrm{mAP}}_{50:95}$ standard deviation of 0.0267, indicating stronger sensitivity to initialization on this dataset. These results therefore support good repeatability on VisDrone and UAVDT while showing that robustness is dataset dependent rather than uniform.

#### 6.1.2. Comparison with the YOLOv8n Baseline

The second analysis compares RIF-YOLO-N with the original YOLOv8n baseline under the same dataset splits and validation settings. This controlled comparison isolates the effect of the proposed feature-enhancement design and resolution-guided fine-tuning from differences in training or evaluation protocols.

To examine the sensitivity of the reported results to random initialization, a matched reproducibility experiment is conducted on VisDrone using three independent seeds, {5,42,123}. For each seed, YOLOv8n and RIF-YOLO-N are trained for 100 epochs at 640×640. The RIF-YOLO-N+RGFT configuration then continues from the corresponding RIF checkpoint for an additional 50 epochs at 832×832. All configurations use identical data partitions and matched training settings within this reproducibility experiment, and the resulting checkpoints are evaluated on the same VisDrone validation split at 1024×1024. Table 9 reports the mean ± sample standard deviation across the three independent runs.

Table 9 shows low run-to-run variation across all three configurations. The complete RIF-YOLO-N+RGFT framework achieves the highest mean precision, ${\mathrm{mAP}}_{50}$, and ${\mathrm{mAP}}_{50:95}$, reaching 0.5040±0.0091, 0.4110±0.0006, and 0.2410±0.0005, respectively. RIF-YOLO-N without RGFT achieves the highest recall, 0.4229±0.0015, and improves ${\mathrm{mAP}}_{50:95}$ over YOLOv8n from 0.2311±0.0012 to 0.2364±0.0021, although its mean ${\mathrm{mAP}}_{50}$ decreases from 0.4082±0.0030 to 0.3980±0.0032. This repeated-run analysis, therefore, confirms that the small single-run ${\mathrm{mAP}}_{50}$ difference between YOLOv8n and RIF-YOLO-N without RGFT should not be interpreted as a consistent standalone gain. In contrast, the complete RIF-YOLO-N+RGFT configuration provides the highest mean AP values with low variability across the tested seeds, supporting the reproducibility of the final configuration.

##### VisDrone

Table 10 first summarizes the architectural and computational differences between YOLOv8n and RIF-YOLO-N. The proposed model retains the original P3/P4/P5 detection structure while introducing identity-safe P3 feature enhancement and resolution-guided fine-tuning. This modification increases the parameter count from 3.008M to 3.061M and the computational cost from 8.1 to 8.8 GFLOPs. At 1024×1024, the controlled forward-pass benchmark increases the mean latency from 18.272 ms for YOLOv8n to 19.035 ms for RIF-YOLO-N, corresponding to an additional 0.763 ms or 4.17%.

**Figure 5:**
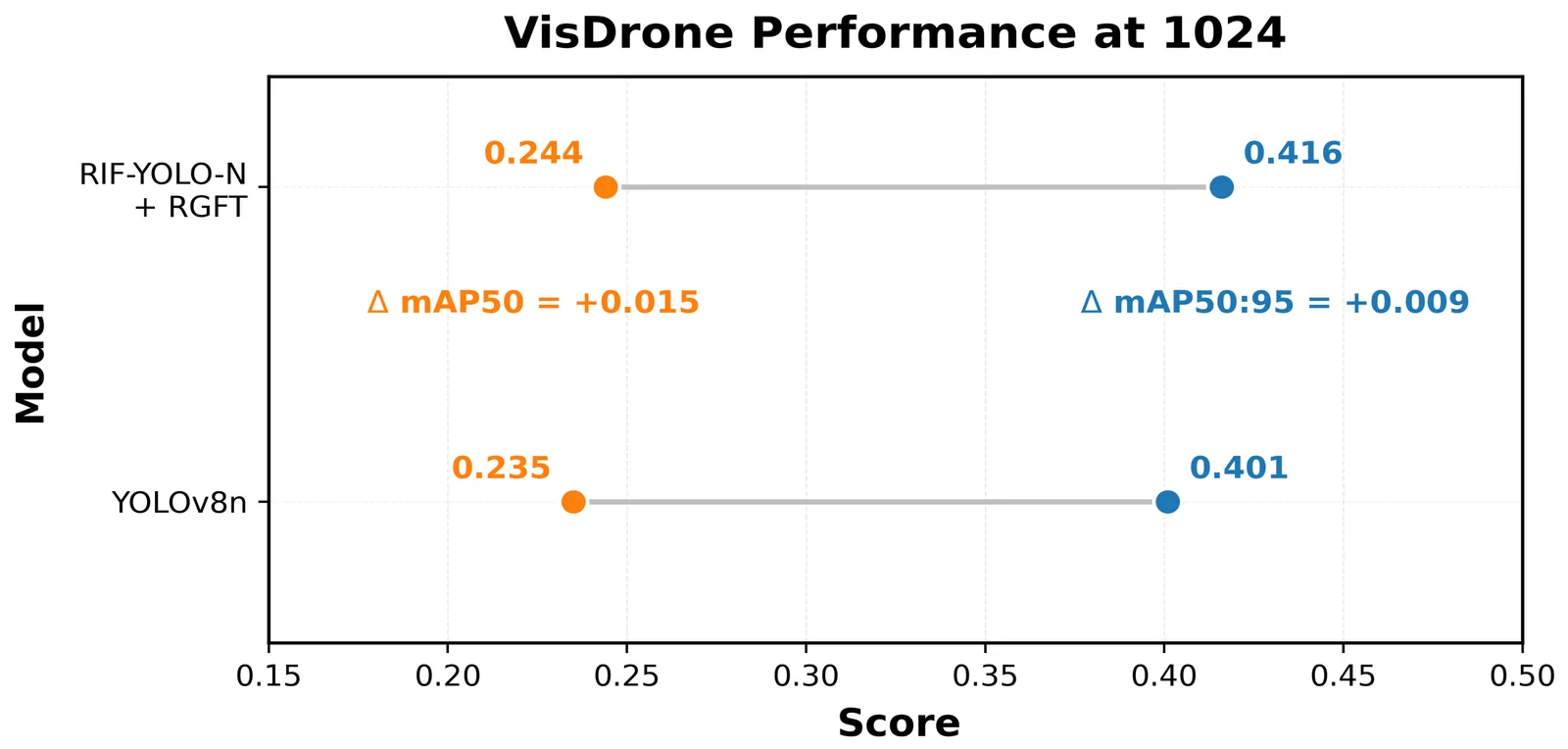
Controlled mAP comparison between YOLOv8n and RIF-YOLO-N on VisDrone at 1024×1024.

Figure 5 presents the corresponding detection performance at 1024×1024, where RIF-YOLO-N achieves its strongest overall VisDrone result. The proposed model increases ${\mathrm{mAP}}_{50}$ from 0.401 to 0.415 and ${\mathrm{mAP}}_{50:95}$ from 0.235 to 0.243. These improvements are achieved with the limited computational increase reported in Table 10, showing that the proposed P3 enhancement improves the controlled YOLOv8n baseline without introducing an additional detection scale.

To examine whether this improvement remains consistent across different input sizes, Table 11 extends the controlled comparison over five validation resolutions. The table also separates RIF-YOLO-N without resolution-guided fine-tuning from the final RIF-YOLO-N + RGFT configuration, allowing the architectural modification and the fine-tuning stage to be assessed independently.

The resolutions reported in Table 11 represent evaluation-time input resolutions for the selected model checkpoints and should not be interpreted as an exhaustive search over RGFT training resolutions. The final VisDrone RGFT checkpoint was fine-tuned at 832×832 and then evaluated across resolutions from 640×640 to 1024×1024 to measure its sensitivity to operating resolution.

The resolution analysis shows that both detectors benefit from increased input resolution, but the performance margin of RIF-YOLO-N becomes more evident at the higher resolutions. At 832×832, 960×960, and 1024×1024, the final RIF-YOLO-N achieves ${\mathrm{mAP}}_{50}$ values of 0.381, 0.401, and 0.415, respectively, compared with 0.374, 0.389, and 0.401 for YOLOv8n. At 1024×1024, the final model also improves precision from 0.507 to 0.520, recall from 0.411 to 0.424, and ${\mathrm{mAP}}_{50:95}$ from 0.235 to 0.243.

The VisDrone results show that RGFT primarily benefits higher-resolution operation. At 1024×1024, RIF-YOLO-N improves from 0.402/0.232 ${\mathrm{mAP}}_{50}{\mathrm{/mAP}}_{50:95}$ without RGFT to 0.415/0.243 with RGFT, whereas at 640×640 the non-RGFT model is marginally better in ${\mathrm{mAP}}_{50}$ (0.330 versus 0.328). To isolate the effect of the fine-tuning strategy, YOLOv8n was also subjected to the same 640→832 RGFT procedure. At 1024×1024, YOLOv8n + RGFT achieves P=0.511, R=0.415, ${\mathrm{mAP}}_{50}$ = 0.405, and ${\mathrm{mAP}}_{50:95}$ = 0.238, while RIF-YOLO-N + RGFT reaches 0.520, 0.424, 0.415, and 0.243, respectively. Thus, under the same RGFT protocol, RIF provides additional gains of 0.009 in precision and recall, 0.010 in ${\mathrm{mAP}}_{50}$, and 0.005 in ${\mathrm{mAP}}_{50:95}$. These results indicate that RIF and RGFT are complementary, with RGFT mainly improving high-resolution performance rather than producing uniform gains across all input sizes.

The VisDrone results separate the effects of RGFT and RIF more explicitly. At 1024×1024, applying the same 640→832 RGFT procedure to YOLOv8n improves ${\mathrm{mAP}}_{50}{\mathrm{/mAP}}_{50:95}$ from 0.401/0.235 to 0.405/0.238, isolating the gain associated with RGFT alone. Under this matched RGFT protocol, RIF-YOLO-N + RGFT reaches 0.415/0.243, providing additional gains of 0.010 and 0.005 over YOLOv8n + RGFT. RIF-YOLO-N without RGFT achieves 0.402/0.232, showing that the architectural modification does not independently improve all metrics. These results therefore indicate complementary rather than uniformly additive effects: RGFT provides a small baseline gain, while RIF contributes additional improvement when both detectors undergo the same high-resolution fine-tuning procedure.

##### UAVDT

Table 12 reports the controlled UAVDT comparison. At 640×640, RIF-YOLO-N + RGFT increases precision from 0.871 to 0.882, recall from 0.791 to 0.814, and ${\mathrm{mAP}}_{50}$ from 0.853 to 0.870 relative to YOLOv8n. At 1024×1024, the proposed model increases recall from 0.814 to 0.838, ${\mathrm{mAP}}_{50}$ from 0.833 to 0.862, and ${\mathrm{mAP}}_{50:95}$ from 0.519 to 0.543.

**Figure 6:**
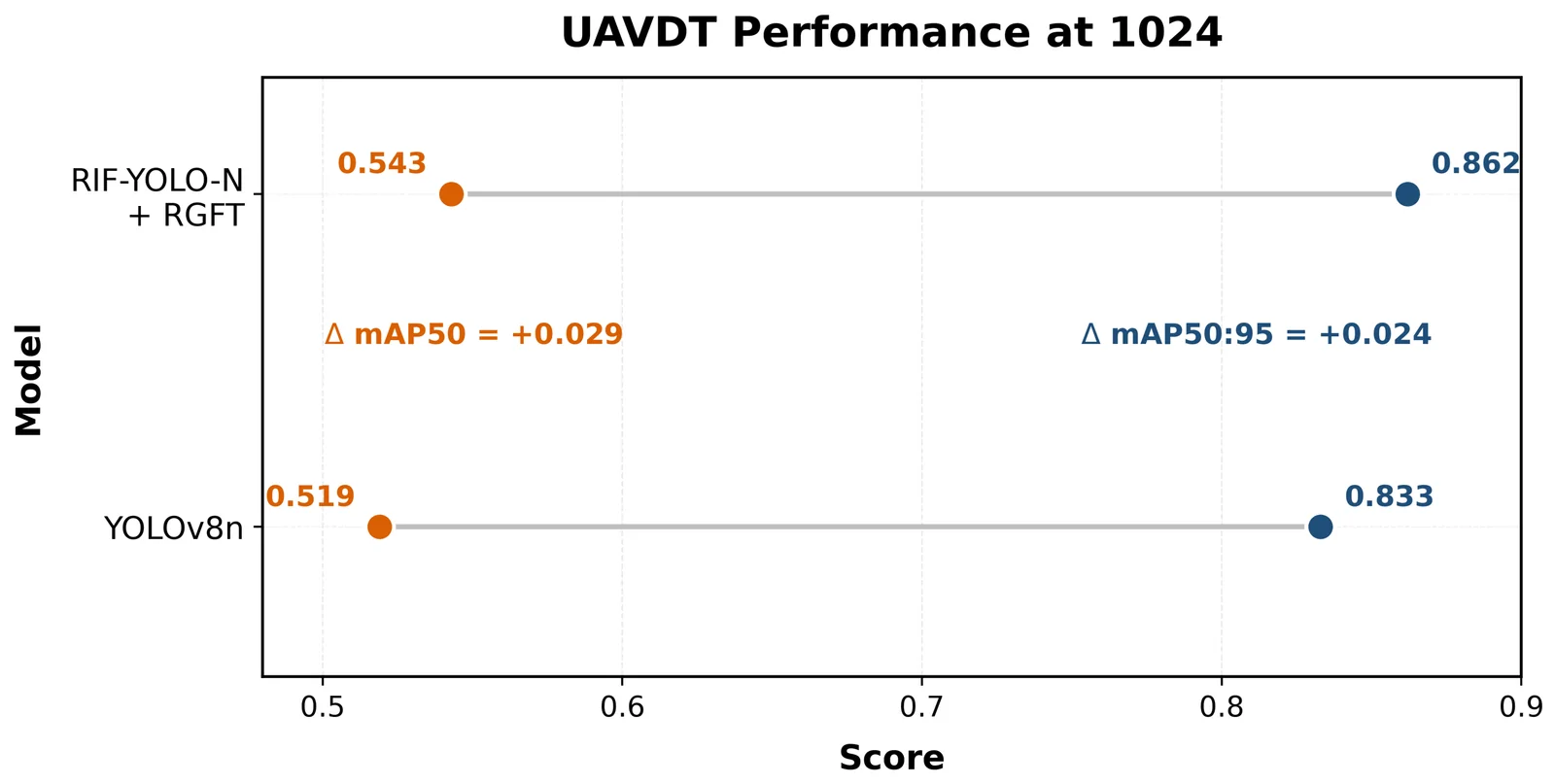
Controlled mAP comparison between YOLOv8n and RIF-YOLO-N on UAVDT at 1024×1024.

The UAVDT results show that RGFT plays an important role in the final external-dataset behavior. The highest ${\mathrm{mAP}}_{50}$ occurs at 640×640, while the highest ${\mathrm{mAP}}_{50:95}$ occurs at 1024×1024. This differs from VisDrone, where both metrics reach their highest values at the largest evaluated resolution. The result indicates that the effect of input resolution depends on the dataset rather than following a single monotonic operating rule across aerial benchmarks. Figure 6 provides a direct visual comparison of ${\mathrm{mAP}}_{50}$ and ${\mathrm{mAP}}_{50:95}$ at 1024×1024.

##### VEDAI

Table 13 reports the controlled comparison on the VEDAI test set. YOLOv8n and RIF-YOLO-N without RGFT are evaluated at multiple input resolutions to examine their sensitivity to image scale. For RGFT, separate resolution-matched checkpoints are fine-tuned and evaluated at 832×832 and 1024×1024. This additional experiment examines whether the 832×832 target selected for VisDrone and UAVDT transfers directly to a different aerial dataset or whether the effective RGFT operating resolution depends on the dataset.

The VEDAI results show a strongly non-monotonic response to input resolution. At 832×832, RIF-YOLO-N + RGFT improves ${\mathrm{mAP}}_{50}$ and ${\mathrm{mAP}}_{50:95}$ from 0.573 and 0.355 for YOLOv8n to 0.716 and 0.427, corresponding to absolute gains of 0.143 and 0.072, respectively. At 1024×1024, the resolution-matched RGFT-1024 model achieves P=0.799, R=0.586, ${\mathrm{mAP}}_{50}$ = 0.669, and ${\mathrm{mAP}}_{50:95}$ = 0.419, exceeding YOLOv8n at the same resolution by 0.260 and 0.167 in ${\mathrm{mAP}}_{50}$ and ${\mathrm{mAP}}_{50:95}$, respectively. The non-monotonic behavior, however, cannot be attributed only to the selected RGFT target resolution because substantial degradation at 1024×1024 is also observed for YOLOv8n and RIF-YOLO-N without RGFT. For YOLOv8n, increasing the evaluation resolution from 832×832 to 1024×1024 reduces precision from 0.582 to 0.387 and decreases ${\mathrm{mAP}}_{50}$ from 0.573 to 0.409, whereas RIF-YOLO-N without RGFT shows a stronger reduction in recall from 0.484 to 0.391 together with a decrease in ${\mathrm{mAP}}_{50}$ from 0.633 to 0.414. These different error patterns indicate that higher-resolution evaluation does not produce a uniform effect across detector configurations and can alter the balance between false-positive and missed detections when the operating scale differs from the condition under which the checkpoint was optimized.

**Figure 7:**
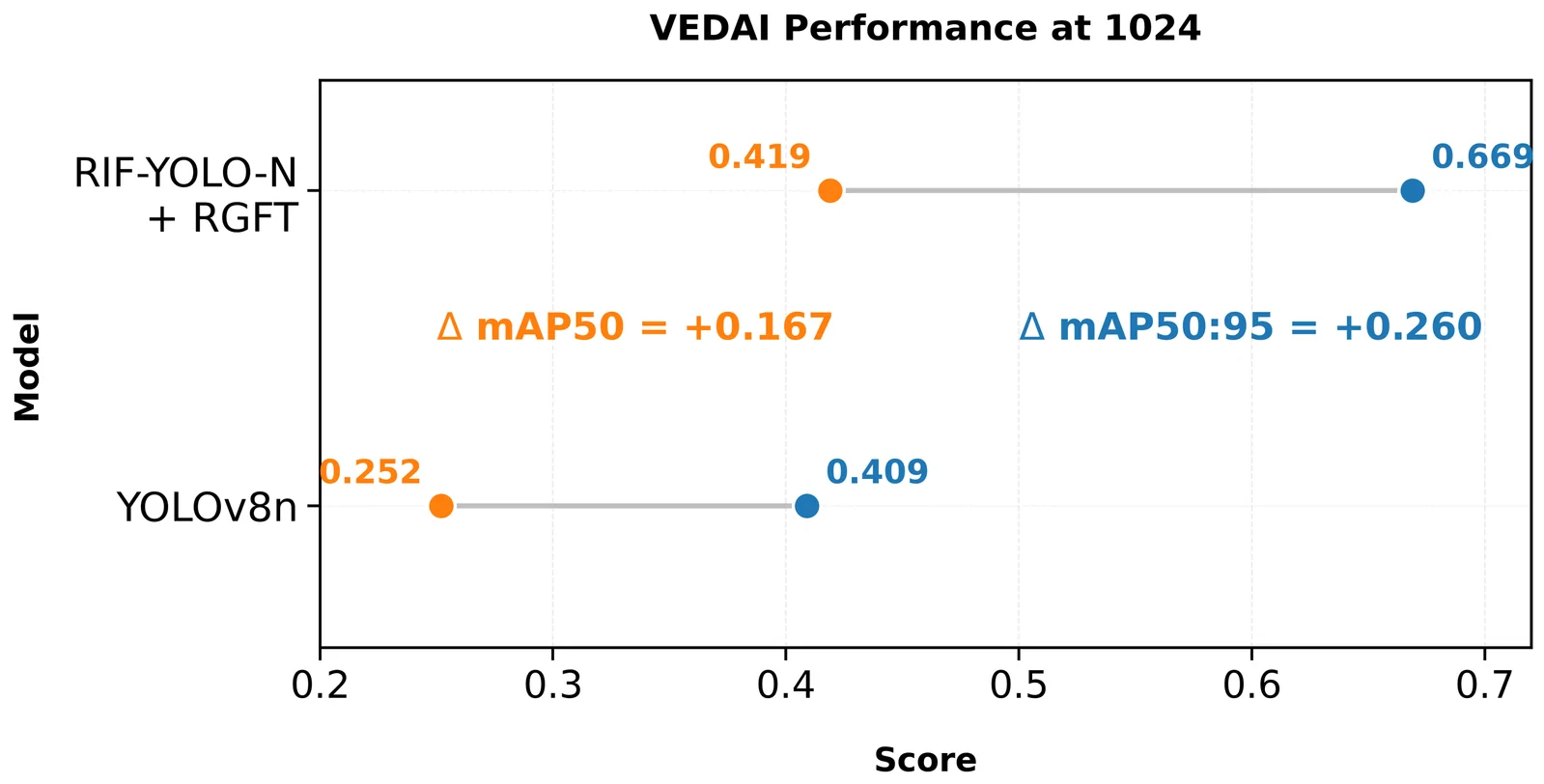
Controlled mAP comparison between YOLOv8n and RIF-YOLO-N on VEDAI at 1024×1024.

The resolution-matched RGFT checkpoints show a different pattern: the 1024×1024 model achieves slightly higher precision and recall than the 832×832 model, increasing from 0.788 and 0.558 to 0.799 and 0.586, respectively, while ${\mathrm{mAP}}_{50}$ decreases from 0.716 to 0.669 and ${\mathrm{mAP}}_{50:95}$ decreases from 0.427 to 0.419. This difference is consistent with the fact that the reported precision and recall represent a selected operating point, whereas average precision summarizes the complete precision–recall curve across confidence thresholds. The relatively small VEDAI test set, containing 365 instances distributed across nine classes, can also make macro-averaged AP more sensitive to class-specific performance changes. The comparatively large VEDAI gain should therefore be interpreted as dataset-specific evidence rather than as uniform cross-dataset generalization. Differences in object distribution, sample count, class composition, and the conversion of VEDAI’s oriented annotations to axis-aligned bounding boxes may be associated with the observed response, but the present experiments do not isolate the individual effects of these factors and therefore do not establish causality. Thus, consistent with the RGFT formulation introduced in Section 4.6, the target resolution $s_{h}$ remains an empirical dataset-dependent hyperparameter, but the observed VEDAI non-monotonicity should be interpreted more broadly as an interaction between training–evaluation scale, model-specific precision–recall behavior, and dataset-level sensitivity rather than as a consequence of resolution-specific adaptation alone. For consistency, the 1024×1024 comparison corresponds to ${\mathrm{mAP}}_{50}$ increasing from 0.409 to 0.669, an absolute gain of 0.260, and ${\mathrm{mAP}}_{50:95}$ increasing from 0.252 to 0.419, an absolute gain of 0.167. Figure 7 visualizes this controlled comparison.

#### 6.1.3. Contextual Comparison with Representative Aerial Object Detectors

The following comparison provides contextual positioning of RIF-YOLO-N relative to representative aerial small-object detectors reported in the literature. The selected studies include lightweight detectors, high-resolution architectures, and cluster-based methods for tiny-object detection. These cross-study results are not protocol-matched because the reported methods differ in input resolution, dataset splits, training schedules, augmentation strategies, implementation frameworks, inference procedures, and hardware platforms. Accordingly, the literature values are used only to describe architectural and computational context and are not interpreted as a controlled ranking or as direct evidence of superiority. The primary comparative evidence for RIF-YOLO-N is instead provided by the controlled YOLOv8n comparisons reported in Table 11, Table 12 and Table 13, where the baseline and proposed configurations use matched dataset splits and evaluation protocols. In particular, the VisDrone comparison in Table 11 additionally includes YOLOv8n + RGFT under the same 640→832 fine-tuning procedure as RIF-YOLO-N + RGFT, providing a unified training-strategy control for separating architectural and fine-tuning effects.

Table 14 summarizes the main architectural differences. LEAF-YOLO and TPH-YOLOv5 employ additional high-resolution detection behavior to strengthen small-object representation. YOLC detects dense object clusters and applies zoom-in processing before final detection. EdgeYOLO targets edge-oriented real-time detection. RIF-YOLO-N instead retains the original YOLOv8n P3/P4/P5 detection hierarchy and modifies the P3 feature pathway through identity-safe residual enhancement and resolution-guided fine-tuning.

Table 15 summarizes the model complexity and inference information reported in the corresponding studies. Because these values were obtained using different input resolutions, hardware platforms, software implementations, and timing protocols, they are presented as descriptive computational context and are not used for direct efficiency ranking.

Table 16 summarizes the detection results reported for the selected aerial object detectors. AP denotes ${\mathrm{mAP}}_{50:95}$ and ${\mathrm{AP}}_{50}$ denotes mAP at an IoU threshold of 0.5. The entries are reproduced as literature-reported reference values; differences in input resolution, dataset splits, training schedules, augmentations, and evaluation settings prevent direct quantitative ranking across methods.

Table 16 shows that the representative methods adopt substantially different architectural and computational strategies, including additional high-resolution prediction scales, cluster-based zooming, transformer-enhanced prediction, and lightweight edge-oriented designs. Because the corresponding results were obtained under different experimental protocols, numerical differences in AP, ${\mathrm{AP}}_{50}$, model complexity, or inference speed should not be interpreted as direct evidence that one method outperforms another. The literature comparison is therefore used only to position RIF-YOLO-N within the broader design space of aerial small-object detection. The effectiveness of the proposed framework is assessed primarily through the controlled YOLOv8n comparisons on VisDrone, UAVDT, and VEDAI, where the baseline and proposed models are evaluated under matched dataset splits and validation settings. Under these controlled conditions, RIF-YOLO-N preserves the original P3/P4/P5 prediction hierarchy while introducing targeted P3 enhancement and resolution-guided fine-tuning, enabling evaluation of the proposed design without relying on cross-study ranking.

The controlled runtime benchmark was performed at 1024×1024 with batch size one on an NVIDIA RTX 2000 Ada Generation Laptop GPU. Each model underwent 50 warm-up iterations followed by 200 timed forward passes. GPU execution was synchronized for every timed iteration before recording latency, and the same procedure was applied to YOLOv8n and RIF-YOLO-N. The reported mean ± standard deviation therefore characterizes model forward-pass latency only; data loading, pre-processing, post-processing, and visualization are outside this timing scope. This controlled YOLOv8n–RIF-YOLO-N pair is therefore used to quantify the runtime overhead introduced by the proposed architecture. Runtime values for the external methods are retained only as literature-reported computational context and are not used for direct speed ranking, as their hardware, input resolutions, software environments, and timing protocols do not match the present benchmark.

Table 17 reports the computational characteristics of the selected detectors. Under the controlled 1024×1024 evaluation, RIF-YOLO-N increases mean latency from 18.272 to 19.035 ms, corresponding to only 0.763 ms or 4.17% overhead, while retaining 52.54 FPS. Because YOLOv8n and RIF-YOLO-N were measured on the same hardware and using the same timing protocol, the overhead is directly interpretable. RGFT adds no inference-time computation because it is applied only during training. Accordingly, no claim is made that RIF-YOLO-N is faster than the externally reported detectors in Table 15, since those methods were not re-benchmarked on the same hardware and under the same runtime protocol. The measured throughput is real-time-capable on the evaluated NVIDIA RTX 2000 Ada Generation Laptop GPU, but performance on resource-constrained onboard UAV hardware remains hardware-dependent and requires dedicated embedded-device validation. Runtime values reported for external methods are retained only for the literature context, as their hardware and evaluation protocols differ.

### 6.2. Fine-Grained Tiny-Object and Class-Level Analysis

The primary VisDrone results show the overall gain of RIF-YOLO-N, but tiny-object detection requires a finer analysis because the benchmark is highly imbalanced across object scales and classes. This subsection analyzes the validation set from two complementary perspectives: object-size distribution and class-wise AP behavior. The objective is to determine whether the reported improvement is aligned with the target problem rather than being caused only by dominant large or easy categories.

Table 18 reports the object-size distribution of the VisDrone validation set. The bounding boxes are grouped using normalized area *A*. Tiny objects account for 25,967 instances, and small objects account for 11,894 instances. Together, these two groups represent 97.69% of all validation objects. This distribution confirms that the dataset is dominated by tiny and small targets, so improvements in these groups provide the most relevant evidence for the proposed framework.

Table 19 presents the size-stratified ${\mathrm{AP}}_{50}$ comparison for the dominant object groups. RIF-YOLO-N improves ${\mathrm{AP}}_{50}$ from 0.1298 to 0.1346 for tiny objects and from 0.3771 to 0.3852 for small objects, corresponding to absolute gains of 0.0048 and 0.0081, respectively. The larger improvement is therefore observed for the small-object group, while the gain for the tiniest objects remains positive but limited. This suggests that the enhanced P3 pathway is more effective when the target retains sufficient spatial structure for localization. Extremely tiny objects remain challenging because they may occupy only a few pixels after resizing and are often affected by crowding, occlusion, and weak contrast.

**Figure 8:**
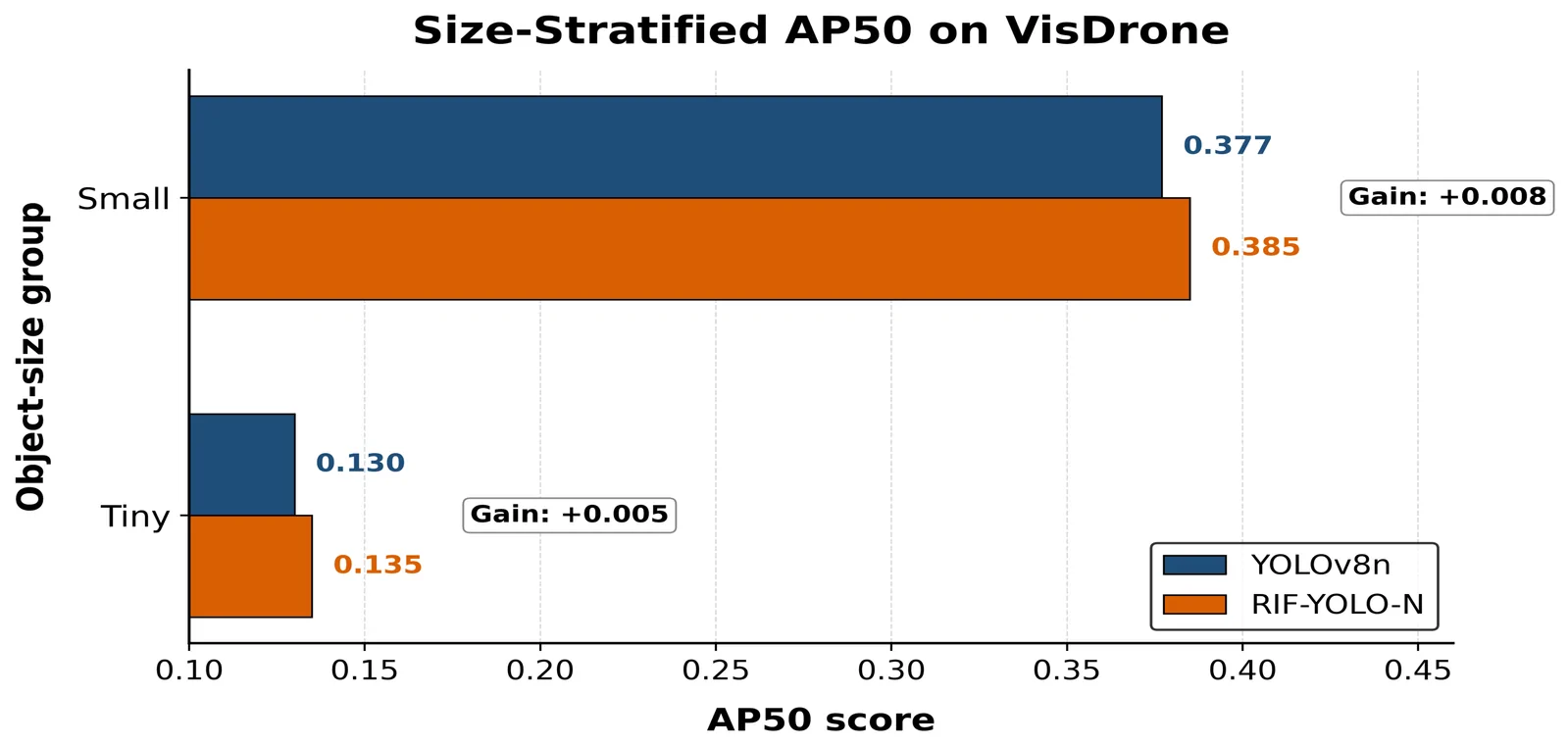
Size-stratified ${\mathrm{AP}}_{50}$ comparison between YOLOv8n and RIF-YOLO-N on the VisDrone validation set.

Figure 8 gives the same size-stratified result in visual form. The bar graph shows that the proposed model improves both reported object-size groups, while the stronger gain on small objects indicates that the feature enhancement benefits targets that still retain distinguishable local structure.

Class-level behavior was then examined to identify which object categories benefit from the proposed framework. Table 20 reports ${\mathrm{AP}}_{50}$ and ${\mathrm{AP}}_{50:95}$ for each VisDrone category at 1024×1024. RIF-YOLO-N improves ${\mathrm{AP}}_{50}$ in seven out of ten classes. The largest ${\mathrm{AP}}_{50}$ gains occur for van (+0.032), bicycle (+0.031), truck (+0.031), and people (+0.027). The largest ${\mathrm{AP}}_{50:95}$ gains occur for truck (+0.026), van (+0.025), and bicycle (+0.014). These improvements indicate that the proposed feature enhancement helps several small, dense, or visually weak categories.

**Figure 9:**
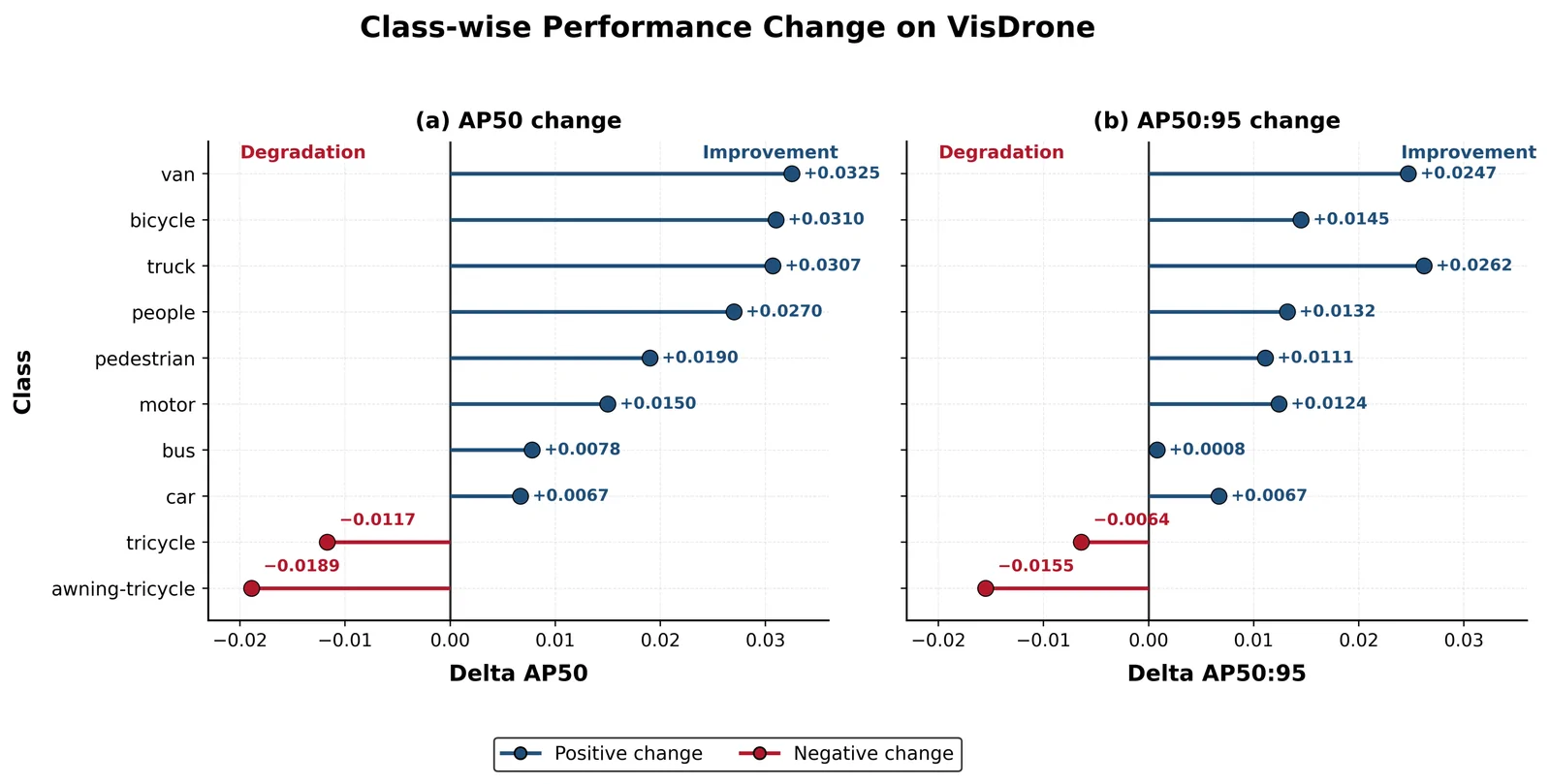
Class-wise performance change of RIF-YOLO-N relative to YOLOv8n on VisDrone. (**a**) AP50 change and (**b**) AP50:95 change. Positive values indicate class-wise improvement, while negative values indicate degradation.

Figure 9 visualizes the class-wise changes. The ${\mathrm{AP}}_{50}$ gain is positive for most categories, which confirms that the overall improvement is not limited to one dominant class. The ${\mathrm{AP}}_{50:95}$ trend is also mostly positive, although the gains are smaller because stricter IoU thresholds require more accurate localization. The negative changes for tricycle and awning-tricycle show that class ambiguity remains a limitation. These two categories are visually similar and have fewer instances than dominant classes such as car, pedestrian, and people. Therefore, the proposed framework improves spatial feature representation, but it does not directly solve rare-class imbalance or inter-class confusion.

Overall, the fine-grained analysis supports the main objective of RIF-YOLO-N. The proposed model improves ${\mathrm{AP}}_{50}$ for both tiny and small object groups and improves most VisDrone categories at the class level. The strongest gains occur in categories where small object scale and local spatial detail are important. Remaining weaknesses occur in visually ambiguous and low-frequency classes, which suggests that future extensions should consider class-rebalancing, ambiguity-aware learning, or category-specific error reduction in addition to lightweight feature enhancement.

### 6.3. Detection Diagnostics and Qualitative Assessment

The previous subsection analyzed object-scale and class-wise AP behavior. This subsection further examines the detection process through diagnostic curves, confusion patterns, and qualitative validation examples. These outputs help explain whether the numerical gains of RIF-YOLO-N are supported by stable confidence behavior and visually meaningful object recovery in dense aerial scenes.

Table 21 summarizes the diagnostic outputs used in this analysis. The precision–recall curve evaluates the trade-off between correct detections and missed objects across confidence thresholds. The F1-confidence curve identifies the confidence region where precision and recall are balanced. The normalized confusion matrix shows category-level prediction errors, including confusion with the background class. Qualitative prediction images provide visual evidence of model behavior in dense tiny-object scenes.

**Figure 10:**
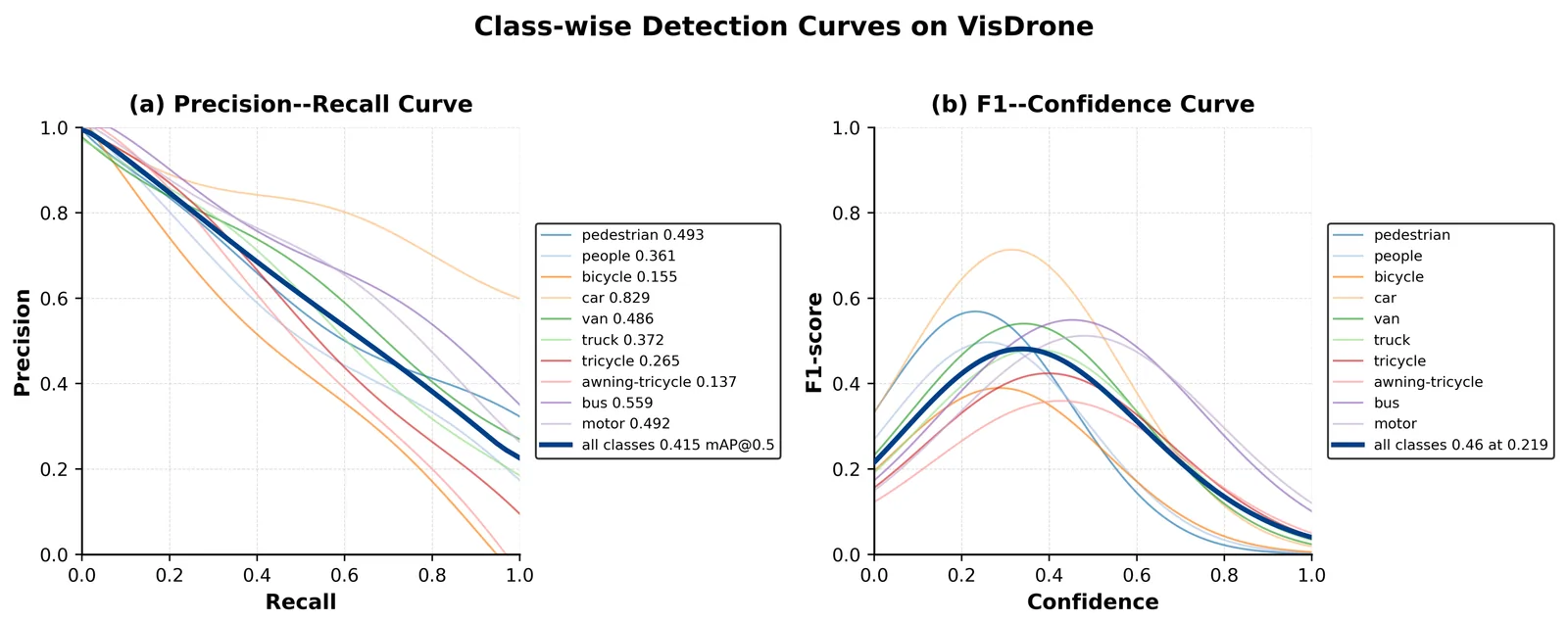
Detection performance of RIF-YOLO-N on the VisDrone validation set at 1024×1024: (**a**) precision–recall curves for individual object classes and the overall detector; (**b**) F1-score versus confidence-threshold curves for individual object classes and the overall detector.

Figure 10 presents the precision–recall and F1-confidence curves for the final RIF-YOLO-N model on the VisDrone validation set at 1024×1024. The precision–recall curve supports the reported AP values by showing the detector behavior over different confidence thresholds rather than at one operating point only. The F1-confidence curve complements this analysis by showing how the model balances false positives and missed detections. This is important for tiny-object detection because overly strict confidence thresholds can suppress weak small-object detections, while overly permissive thresholds can increase background false positives.

**Figure 11:**
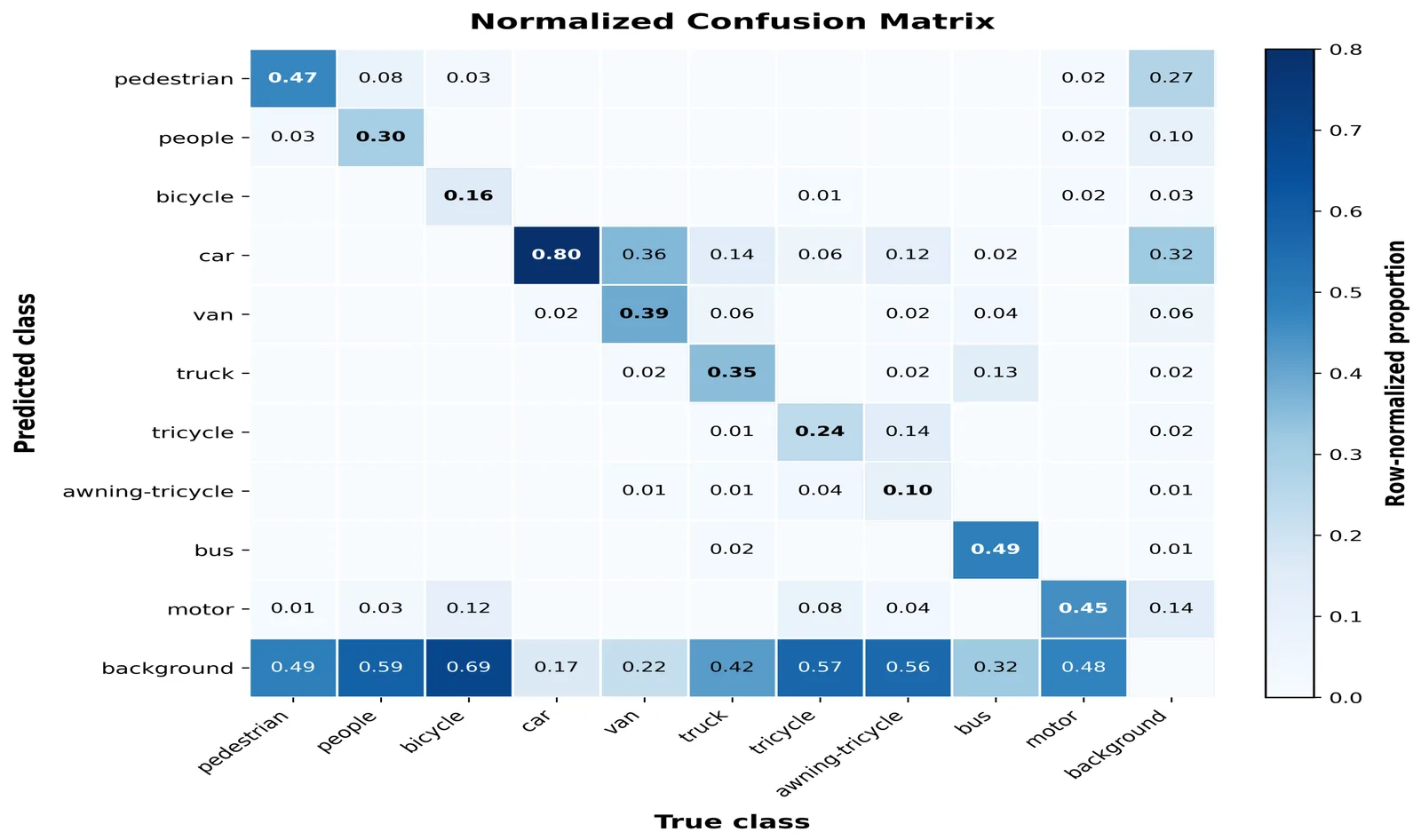
Normalized confusion matrix of RIF-YOLO-N on the VisDrone validation set at 1024×1024.

Figure 11 shows the normalized confusion matrix of RIF-YOLO-N at 1024×1024. The matrix provides a class-level view of detection errors and complements the AP-based class-wise analysis. The main diagonal represents correct class predictions, while off-diagonal entries indicate inter-class confusion. This diagnostic is useful for interpreting the weaker behavior observed for ambiguous categories. In particular, visually related vehicle classes and small human-related categories can be confused under occlusion, low resolution, or dense object placement. The background column and row also help identify missed detections and false positives, which are common error sources in aerial tiny-object detection.

**Figure 12:**
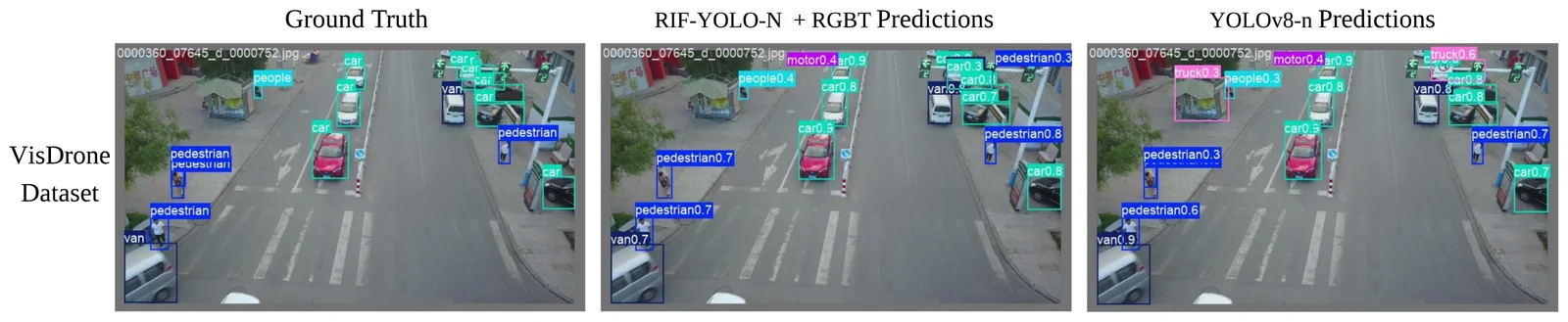
Qualitative prediction comparison on a representative VisDrone validation sample at 1024×1024.

Qualitative prediction samples in Figure 12 compare YOLOv8n and RIF-YOLO-N on the same VisDrone validation scenes at 1024×1024. Although RIF-YOLO-N improves small-object recovery, typical failures remain for extremely small or partially occluded objects, densely overlapping targets, and visually similar classes. Background clutter and low-contrast regions can further weaken object–background separation, leading to missed detections or false positives when background structures resemble target features. These observations are consistent with the off-diagonal and background errors in Figure 11 and indicate that P3 feature enhancement improves fine-scale representation but does not fully resolve ambiguity caused by limited object pixels, occlusion, class similarity, and complex backgrounds.

Overall, the diagnostic analysis complements the aggregate mAP results by characterizing confidence behavior, class confusion, missed detections, background errors, and qualitative failure modes. The remaining errors show that the main limitations arise under extreme object scale, occlusion, dense overlap, visual similarity, and complex background conditions.

### 6.4. Ablation Study of the Proposed RIF-YOLO-N Framework

Ablation experiments were conducted to isolate the contribution of the main components of RIF-YOLO-N. The analysis considers four architectural components of the RIF module: feature projection, adaptive gating, residual refinement, and residual scaling. Each architecture-level ablation removes only one component from the complete RIF configuration while keeping the remaining components unchanged. RGFT is evaluated separately because it modifies the training procedure rather than the RIF architecture. All variants were evaluated on the VisDrone validation set at 1024×1024 using the same validation protocol.

Table 22 summarizes the component-level results. YOLOv8n represents the original lightweight detector. The complete RIF configuration without RGFT serves as the architectural reference for the component-removal experiments. The projection, gate, refinement, and residual-scale variants each remove only the component identified in the corresponding row. The final RIF-YOLO-N configuration combines the complete RIF module with RGFT.

The architecture-only comparison shows that the complete RIF module changes detector behavior even without RGFT. Relative to YOLOv8n, the complete RIF configuration increases recall from 0.411 to 0.422 and changes ${\mathrm{mAP}}_{50}$ from 0.401 to 0.402, while ${\mathrm{mAP}}_{50:95}$ changes from 0.235 to 0.232. The effect of RIF alone is therefore small and metric-dependent. This result also shows that the architectural contribution should not be interpreted solely from the 0.001 ${\mathrm{mAP}}_{50}$ difference.

Removing the feature-projection operations decreases recall from 0.422 to 0.411, ${\mathrm{mAP}}_{50}$ from 0.402 to 0.396, and ${\mathrm{mAP}}_{50:95}$ from 0.232 to 0.228. Precision increases slightly from 0.496 to 0.503, which indicates a shift in the precision–recall operating balance rather than uniform degradation across all metrics. The reduction in both AP measures indicates that the learned projections provide useful representations for gate conditioning and residual-correction estimation.

Removing the adaptive gate decreases recall from 0.422 to 0.409, ${\mathrm{mAP}}_{50}$ from 0.402 to 0.394, and ${\mathrm{mAP}}_{50:95}$ from 0.232 to 0.228. This result indicates that adaptive feature weighting helps regulate the contribution of the projected backbone feature before residual refinement.

Removing the refinement operator produces the largest AP reduction among the tested architectural removals. Precision decreases from 0.496 to 0.487, recall decreases from 0.422 to 0.416, ${\mathrm{mAP}}_{50}$ decreases from 0.402 to 0.391, and ${\mathrm{mAP}}_{50:95}$ decreases from 0.232 to 0.226. This result indicates that gated feature selection alone does not provide the complete residual transformation. The 3×3 refinement operator spatially transforms the selected backbone representation before the correction is added to the preserved P3 pathway.

Removing the residual scale also reduces performance. The corresponding variant obtains 0.394 ${\mathrm{mAP}}_{50}$ and 0.227 ${\mathrm{mAP}}_{50:95}$, compared with 0.402 and 0.232 for the complete RIF configuration without RGFT. Precision decreases from 0.496 to 0.485, while recall decreases from 0.422 to 0.416. These results support using the zero-initialized residual scale to control the contribution of the correction branch during optimization.

The final configuration combines the complete RIF module with RGFT. Compared with the complete RIF configuration without RGFT, the final model increases precision from 0.496 to 0.520, recall from 0.422 to 0.424, ${\mathrm{mAP}}_{50}$ from 0.402 to 0.415, and ${\mathrm{mAP}}_{50:95}$ from 0.232 to 0.243. Relative to YOLOv8n, the final configuration improves ${\mathrm{mAP}}_{50}$ from 0.401 to 0.415 and ${\mathrm{mAP}}_{50:95}$ from 0.235 to 0.243.

**Figure 13:**
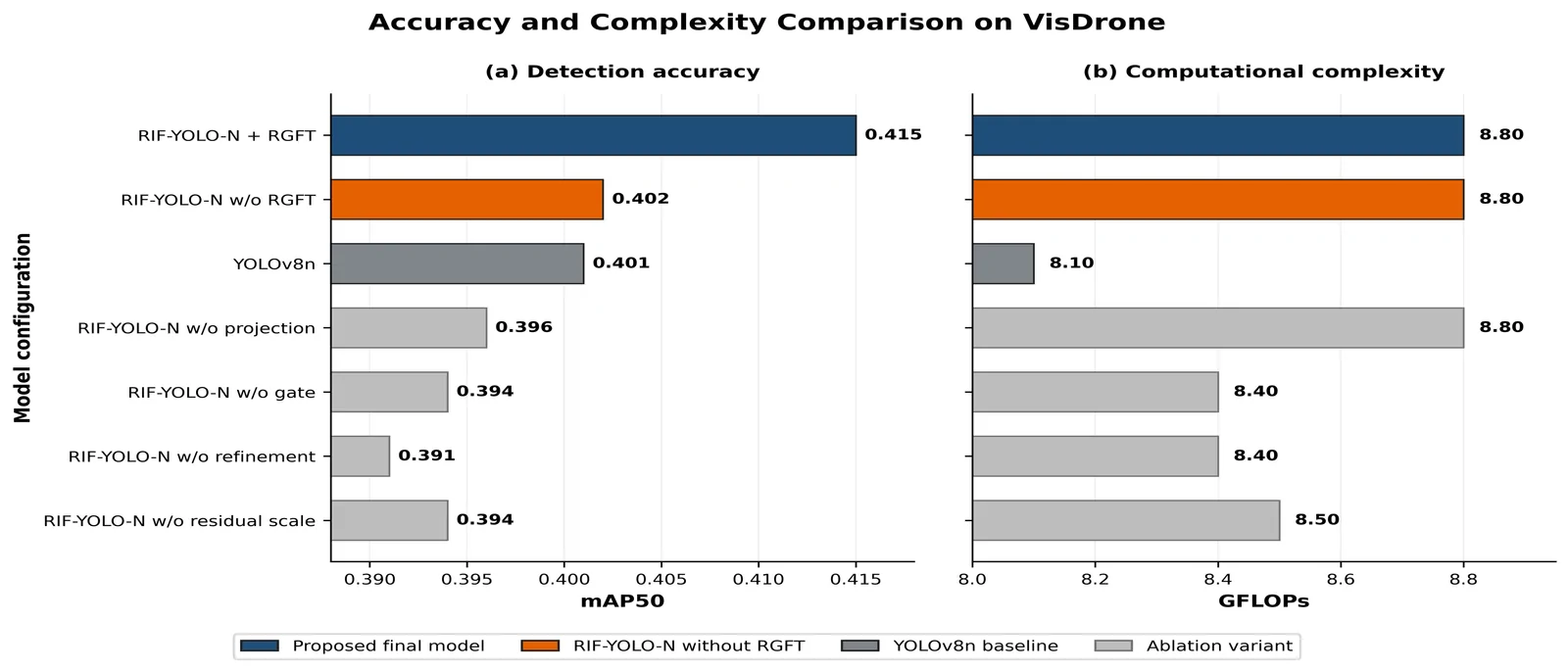
RIF-YOLO-N ablation results on the VisDrone validation set at 1024×1024: (**a**) detection accuracy in terms of ${\mathrm{mAP}}_{50}$; (**b**) computational complexity in terms of GFLOPs.

Among the architecture-level removals, eliminating the refinement operator causes the largest reduction in both AP metrics, while removing the gate, residual scale, or projection also degrades performance relative to the complete RIF configuration without RGFT, indicating that the module benefits from the combined effects of feature projection, adaptive weighting, spatial refinement, and controlled residual injection, whereas RGFT provides an additional training-stage improvement; Figure 13 further shows that the final RIF-YOLO-N achieves the highest ${\mathrm{mAP}}_{50}$ with only a modest increase in computational cost over the YOLOv8n baseline, supporting the use of a lightweight feature-enhancement strategy rather than a heavier detection branch.

### 6.5. Architecture Selection Analysis

Several architecture variants were evaluated during model development to determine whether the final detector should rely on heavier high-resolution processing or a controlled lightweight enhancement of the original YOLOv8n hierarchy. This analysis differs from the ablation study in Section 6.4. The ablation study removes individual components from the proposed framework, whereas the architecture selection analysis compares broader detector designs that modify the feature hierarchy, detection scale, or enhancement strategy. Because these variants were compared during model development using the VisDrone validation split, as they constitute architecture-selection evidence rather than an independent test-set evaluation.

Table 23 summarizes the architecture design space. The final RIF-YOLO-N retains the original P3/P4/P5 detection hierarchy and applies identity-safe enhancement only to the P3 pathway. The alternative designs either introduce P2-level processing, remove the P5 semantic pathway, or add stronger high-resolution context aggregation. This comparison clarifies that the final design was selected after evaluating structurally different detector configurations, not only by modifying a single module.

Table 24 reports the quantitative results of these architecture variants on the VisDrone validation set at 1024×1024. The final RIF-YOLO-N obtains the best overall result, with 0.520 precision, 0.424 recall, 0.415 ${\mathrm{mAP}}_{50}$, and 0.243 ${\mathrm{mAP}}_{50:95}$. The P2/P3/P4 variant without P5 performs substantially worse than the selected P3/P4/P5 configuration, showing that shifting the prediction hierarchy toward a finer P2 scale while omitting P5 does not improve detection. Because this variant simultaneously introduces P2 and removes P5, the observed difference should be interpreted as an architecture-level effect rather than an isolated contribution of P5. The heavy LEAF-style V3 design achieves competitive precision, but it requires much higher computational cost and still remains below the final RIF-YOLO-N in AP. The V3-Efficient model keeps the complexity close to the final model, but its accuracy is lower.

To further clarify the selection decision, Table 25 reports the relative difference between each alternative architecture and the final RIF-YOLO-N. The P2/P3/P4 design loses 0.055 ${\mathrm{mAP}}_{50}$ and 0.034 ${\mathrm{mAP}}_{50:95}$ despite adding a high-resolution detection scale. The heavy LEAF-style V3 model is closer in AP, but it adds 2.098M parameters and 29.8 GFLOPs while still reducing ${\mathrm{mAP}}_{50}$ by 0.011 and ${\mathrm{mAP}}_{50:95}$ by 0.007. The V3-Efficient model has similar parameter count and slightly lower GFLOPs, but it loses 0.032 ${\mathrm{mAP}}_{50}$ and 0.022 ${\mathrm{mAP}}_{50:95}$. These results show that the final model is not only more accurate than the alternatives, but also more efficient than the heavy high-resolution design.

**Figure 14:**
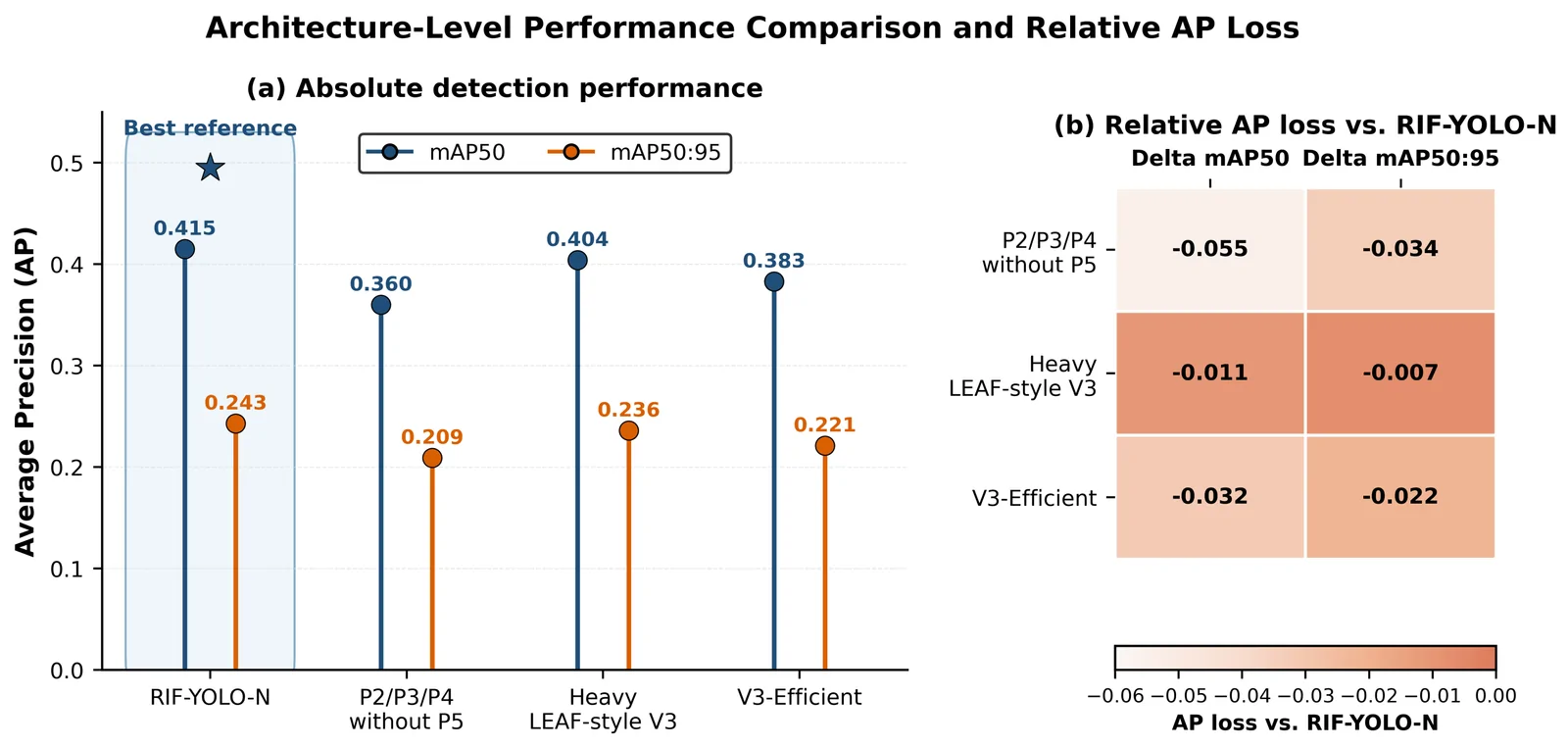
(**a**) absolute detection performance of RIF-YOLO-N and the alternative architectures in terms of ${\mathrm{mAP}}_{50}$ and ${\mathrm{mAP}}_{50:95}$; (**b**) relative ${\mathrm{mAP}}_{50}$ and ${\mathrm{mAP}}_{50:95}$ loss of each alternative architecture with respect to RIF-YOLO-N.

Figure 14 compares the AP values of the architecture variants. The final RIF-YOLO-N achieves the highest ${\mathrm{mAP}}_{50}$ and ${\mathrm{mAP}}_{50:95}$, supporting the selection of the same-scale P3 enhancement over heavier high-resolution alternatives. Figure 14 also shows the AP loss of each alternative architecture relative to the final RIF-YOLO-N. This representation gives a direct ranking of the explored designs and highlights the accuracy gap between the final model and the alternatives.

The architecture selection results show that stronger high-resolution processing does not automatically improve tiny-object detection. The P2/P3/P4 result shows that emphasizing a finer prediction scale while altering the original hierarchy does not outperform the selected P3/P4/P5 design. The heavy LEAF-style V3 result shows that high-resolution context aggregation can remain competitive, but its 38.6 GFLOPs cost is not justified because its AP remains below the final model. The V3-Efficient result shows that lightweight P2 guidance alone is also insufficient.

The final RIF-YOLO-N was therefore selected because it improves the P3 representation while preserving the original anchor-free P3/P4/P5 detection hierarchy. This design keeps the model compact, avoids adding an extra detection head, preserves semantic context, and provides the best observed balance between detection accuracy and computational cost.

### 6.6. Summary of Observed Trends

The results show that RIF-YOLO-N improves YOLOv8n while preserving a lightweight detection structure. On VisDrone, the final model improves precision, recall, ${\mathrm{mAP}}_{50}$, and ${\mathrm{mAP}}_{50:95}$ at 1024×1024 with only 0.053M additional parameters and 0.7 additional GFLOPs. The resolution analysis shows that the benefit of higher input resolution and RGFT is dataset dependent rather than monotonic; the strongest operating point varies across datasets and metrics, and VEDAI achieves slightly higher AP with the 832×832 RGFT configuration than with the 1024×1024 configuration. The size-stratified and class-wise results show that RIF-YOLO-N improves ${\mathrm{AP}}_{50}$ for both tiny and small objects, with the larger gain observed for the small-object group and only a limited improvement for the tiniest objects, while ${\mathrm{AP}}_{50}$ improves in seven of ten VisDrone categories.

The diagnostic, external validation, ablation, and architecture-selection results support the same conclusion. The PR curves, F1-confidence behavior, confusion matrix, and qualitative samples confirm that the model improves object recovery while retaining stable detection behavior. UAVDT validation shows that the proposed framework also improves performance on an external aerial dataset, especially in recall and localization quality at 1024×1024. The VEDAI results show a more complex resolution response than a simple monotonic scale effect. Although resolution-matched RGFT substantially recovers performance at 832×832 and 1024×1024, the degradation observed for YOLOv8n and RIF-YOLO-N without RGFT at the larger input size shows that the behavior cannot be explained by RGFT target selection alone. The results instead indicate an interaction among training–evaluation scale, model-specific precision–recall behavior, and dataset-level sensitivity, while supporting the treatment of $s_{h}$ as an empirical dataset-dependent RGFT hyperparameter. The ablation study confirms the contribution of the gate, identity-safe residual scale, and resolution-guided fine-tuning, while the architecture analysis shows that heavier high-resolution variants do not provide a better accuracy–complexity balance. Remaining limitations include rare and visually ambiguous classes, severe occlusion, extremely small objects, dataset-dependent response magnitude, non-monotonic resolution sensitivity, and hardware-specific deployment constraints. Differences in dataset composition and annotation representation may contribute to the observed cross-dataset variability, although their individual effects are not isolated in the present experiments. The current runtime evaluation was performed on an NVIDIA RTX 2000 Ada Generation Laptop GPU, so the reported 52.54 FPS throughput does not directly establish equivalent real-time performance on power-constrained onboard UAV processors.

**Figure 15:**
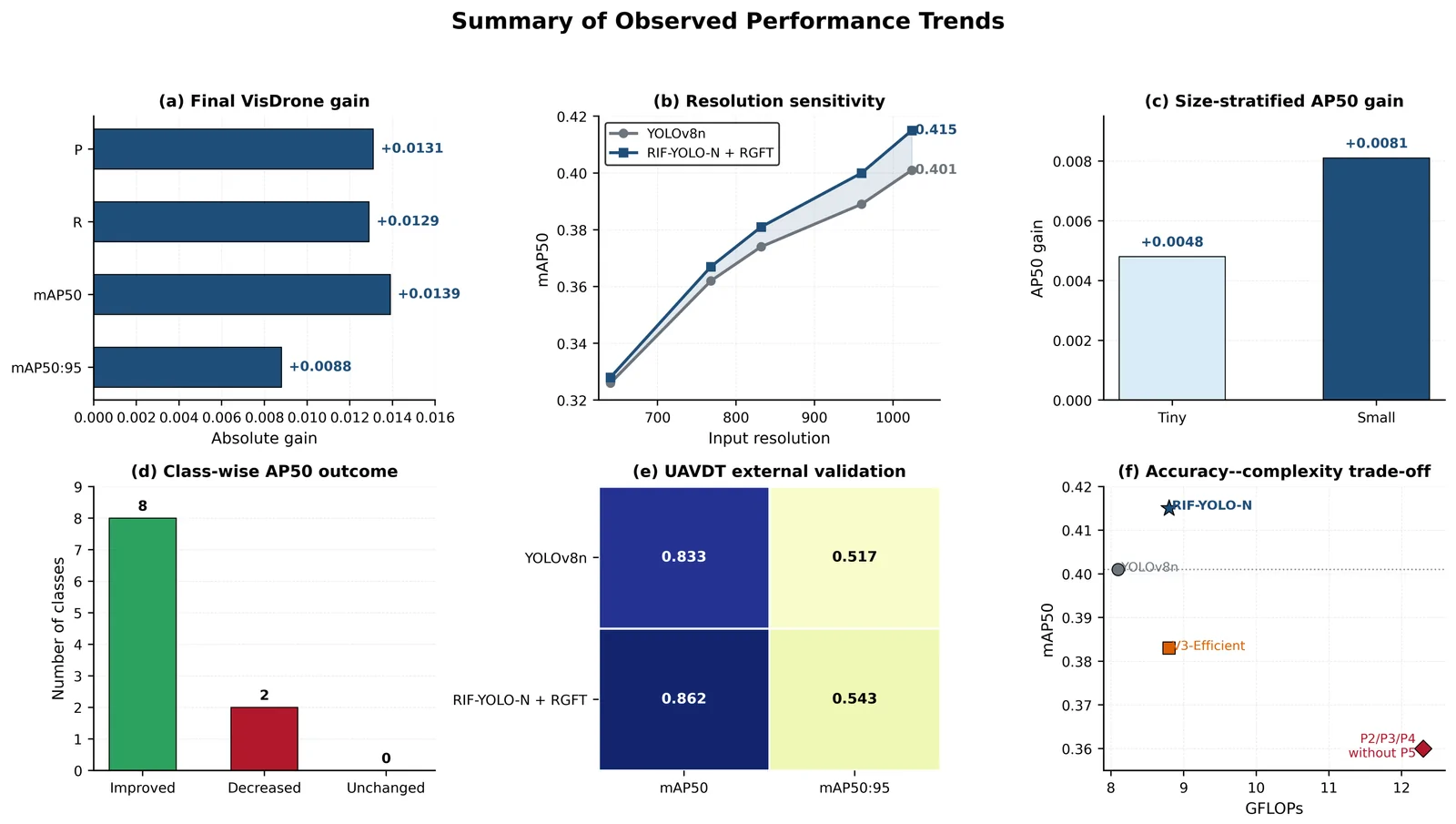
Summary of observed performance trends. (**a**) VisDrone gains over YOLOv8n; (**b**) ${\mathrm{mAP}}_{50}$ across input resolutions; (**c**) size-stratified ${\mathrm{AP}}_{50}$ gains; (**d**) class-wise ${\mathrm{AP}}_{50}$ changes; (**e**) UAVDT external-validation performance; and (**f**) accuracy–complexity trade-off across architectural variants.

Figure 15 summarizes these trends in a single visual panel, combining the main VisDrone gain, resolution behavior, size-stratified improvement, class-wise outcome, UAVDT external validation, and architecture-level accuracy–complexity comparison.

### 6.7. Discussion and Limitations

To enhance the representation of tiny objects, RIF-YOLO-N strengthens the P3 pathway while maintaining the original P3/P4/P5 detection hierarchy. The RIF module integrates semantic information from the neck level and spatial information from the backbone level using an identity-safe residual enhancement mechanism, while the RGFT simply passes the trained detector through the backbone at a higher target resolution without altering the detection network’s structure. The ablation and architecture-selection experiments demonstrate that targeted P3 enhancement provides a favorable accuracy–complexity trade-off compared with heavier high-resolution feature modifications or additional prediction-scale designs.

The experimental results show that the effectiveness of RIF-YOLO-N depends on the dataset and operating resolution. At 1024×1024, the final model improves ${\mathrm{mAP}}_{50}$ and ${\mathrm{mAP}}_{50:95}$ over YOLOv8n by 0.014 and 0.008 on VisDrone, compared with 0.260 and 0.167 on VEDAI. UAVDT also shows metric-dependent resolution behavior. On VEDAI, the 832×832 RGFT configuration reaches ${\mathrm{mAP}}_{50}$ = 0.716 and ${\mathrm{mAP}}_{50:95}$ = 0.427, slightly above the 1024×1024 configuration at 0.669 and 0.419, respectively. These results show that increasing resolution does not produce monotonic AP gains and support treating the RGFT target resolution as an empirical dataset-dependent operating choice rather than a universally beneficial scale. Differences in object distribution, sample count, class composition, and VEDAI’s conversion from oriented to axis-aligned bounding boxes may be associated with the observed cross-dataset differences; however, the present experiments do not isolate these factors, and no causal attribution is made.

The current results reveal two main limitations. First, performance improvements do not occur equally across all object types, and classes of similar objects, such as tricycle and awning-tricycle, are not easily separable when objects are partially occluded and have few pixels. Second, the magnitude of the improvement and the preferred RGFT operating resolution vary across datasets and metrics; therefore, the present cross-dataset results should not be interpreted as evidence of uniform generalization. Future research should focus on class-aware or ambiguity-aware learning, adaptive or multi-resolution fine-tuning, and comparing a unified detector with specialized tiny-object detectors under consistent training and evaluation protocols.

## 7. Conclusions and Future Work

This paper presented RIF-YOLO-N, a lightweight tiny-object detector that strengthens the existing P3 pathway through identity-safe residual fusion while preserving the original anchor-free P3/P4/P5 hierarchy of YOLOv8n. RGFT further adapts the same architecture to higher-resolution inputs without adding inference-time components. At 1024×1024, RIF-YOLO-N with RGFT improves ${\mathrm{mAP}}_{50:95}$ from 0.235 to 0.243 on VisDrone, from 0.519 to 0.543 on UAVDT, and from 0.252 to 0.419 on VEDAI. Size- and class-level analyses show that the VisDrone gains are concentrated mainly on tiny and small objects, while the VEDAI results demonstrate that RGFT performance depends on the selected fine-tuning resolution. The framework remains lightweight, increasing complexity from 3.008M to 3.061M parameters and from 8.1 to 8.8 GFLOPs. Ablation and architecture-selection results support the contribution of the selected P3 enhancement. Overall, the observed gains should be interpreted as operating-condition-specific rather than as evidence of uniform cross-dataset generalization: the improvement on VisDrone at 1024×1024 is modest, the gains on UAVDT and VEDAI are larger, and the higher AP obtained by the 832×832 VEDAI RGFT configuration relative to 1024×1024 shows that the preferred RGFT resolution is dataset dependent.

Future work will investigate class-aware and ambiguity-aware learning to improve difficult and visually similar categories, as well as adaptive or multi-resolution RGFT strategies that reduce sensitivity to a fixed target resolution. Additional studies will examine lightweight integration of RIF with other detector families and unified comparisons with specialized tiny-object detectors under identical training and evaluation protocols. Repeated random-seed experiments and broader external validation would also help quantify the statistical robustness and generalizability of the proposed framework. Future deployment-oriented evaluation will also benchmark RIF-YOLO-N on embedded GPU- and NPU-based platforms to quantify latency, throughput, memory use, and power-related constraints under realistic onboard UAV conditions.

## Figures and Tables

**Table 1:** Qualitative comparison of representative method families for tiny-object detection.

Method Family	Fine-Scale Enhancement	Added P2/High-Res. Scale	Attention/Context	Lightweight Focus	Broader Detector Redesign	Controlled P3 Fusion
Feature-pyramid enhancement [9,19]	✓	△	△	△	△	×
High-resolution/P2 methods [10,12,20,27]	✓	✓	△	△	✓	×
Attention/context methods [24,25,26]	△	×	✓	△	△	×
Lightweight detector redesigns [13,14,15]	△	△	△	✓	✓	×
RIF-YOLO-N	✓	×	×	✓	×	✓

✓: included; ×: not used; △: partial or method-dependent.

**Table 2:** Conceptual operator-level comparison of feature-fusion formulations and RIF-YOLO-N.

Property	Cross-Layer Feature Fusion	Residual Attention	RIF-YOLO-N
Feature relationship	Features from different layers; spatial alignment may be required	Attention applied to a main or transformed feature stream	Backbone and neck P3 features at the same stride-8 scale
Fusion role	Constructs a combined feature representation	Reweights feature responses	Introduces a gated residual correction to the preserved P3 representation
Spatial alignment	Required when feature resolutions differ	Not inherent to the formulation	Not required for the selected P3 inputs
Residual initialization	No explicit identity constraint in the generic formulation	Architecture dependent	Zero-initialized residual correction ($\gamma_{0}=0$); identity shortcut in the final channel-compatible P3 configuration
Prediction hierarchy	Architecture dependent	Architecture dependent	Original P3/P4/P5 hierarchy retained
Design objective	Cross-layer or multi-scale feature aggregation	Adaptive feature emphasis	Controlled fine-scale P3 enhancement

**Table 3:** Theoretical comparison of the existing YOLOv8n prediction levels for tiny-object representation.

Level	Stride	Map at 1024	Relative Object Support	Role in RIF-YOLO-N
P3	8	128×128	1	Enhanced for fine-scale localization
P4	16	64×64	1/4 of P3	Retained for intermediate representation
P5	32	32×32	1/16 of P3	Retained for high-level semantic context

**Table 4:** Summary of object detection datasets used for performance evaluation.

Dataset	Domain	Classes	Used Splits	Primary Evaluation Size	Main Characteristics
VisDrone [28]	Drone-based urban aerial scenes	10	Official train and validation splits	548 images, 38,759 instances	Dense scenes, many tiny and small objects, strong scale variation, occlusion, and background clutter.
UAVDT [29]	UAV-based vehicle detection scenes	3	Train, validation, and test splits	271 images, 7046 instances	Vehicle-focused aerial scenes, strong class imbalance, moving-camera viewpoints, and moderate variation in object density.
VEDAI [30]	Overhead aerial vehicle imagery	9	Train, validation, and test splits	121 test images, 365 instances	High-resolution aerial imagery, multiple vehicle categories, small object instances, and substantial variation in object appearance and orientation.

**Table 5:** Hardware and software environment used for model training and validation.

Component	Specification
Operating system	Windows 10.0.26200
Python version	Python 3.10.20
Deep learning framework	PyTorch 2.11.0+cu128
Detection framework	Ultralytics YOLO 8.4.78
CUDA support	CUDA enabled through PyTorch cu128 build
GPU	NVIDIA RTX 2000 Ada Generation Laptop GPU, 8188 MiB memory
CPU	Intel Core Ultra 7 155H, 22 logical CPUs
RAM	31.51 GB
Storage	Local SSD storage
Main libraries	PyTorch, Ultralytics, OpenCV, NumPy, Pandas, Matplotlib 3.11.2
Primary random seed	42
Deterministic training	Enabled where supported by the framework

**Table 6:** Training, fine-tuning, and validation configuration used for reproducibility.

Setting	Value
Baseline detector	YOLOv8n
Proposed detector	RIF-YOLO-N
Pretrained weights	yolov8n.pt for YOLOv8n-compatible layers
Base training resolution	640×640
Base training epochs	100 epochs
Resolution-guided fine-tuning	Fine-tuning from the best base-stage checkpoint at a higher resolution
RGFT target resolution	832×832 for VisDrone and UAVDT; resolution-matched 832×832 and 1024×1024 configurations for the VEDAI resolution analysis, with 1024×1024 used for the final common-resolution comparison.
Fine-tuning epochs	50 epochs for resolution-guided fine-tuning
Validation resolutions	640×640, 768×768, 832×832, 960×960, and 1024×1024, depending on the experiment
Batch size	Selected according to GPU memory while keeping the baseline and proposed model comparison matched within each dataset
Device	CUDA GPU, device 0
Workers	2 workers for the final controlled experiments unless otherwise stated
Random seed	42 for the reported deterministic runs
Additional repeatability seeds	{0,42,123}, to be used only when repeated-run results are reported
Optimizer and scheduler	Ultralytics YOLO default optimizer and learning-rate schedule under version 8.4.78
Data augmentation	Ultralytics YOLO default augmentation policy under version 8.4.78
Checkpoint selection	Best checkpoint selected according to validation performance during training
Label format	YOLO normalized bounding-box format (c,x,y,w,h)
Metrics	Precision, recall, ${\mathrm{mAP}}_{50}$, ${\mathrm{mAP}}_{50:95}$, parameters, GFLOPs, and inference time

**Table 7:** Performance of the proposed RIF-YOLO-N with RGFT at 1024×1024.

Dataset	Evaluation Split	P	R	${\mathrm{mAP}}_{50}$	${\mathrm{mAP}}_{50:95}$
VisDrone	Validation	0.520	0.424	0.415	0.243
UAVDT	Validation	0.832	0.838	0.862	0.543
VEDAI	Held-out test	0.799	0.586	0.669	0.419

**Table 8:** Three-seed robustness evaluation of the proposed RIF-YOLO-N framework. Values are reported as mean ± sample standard deviation across seeds {5,42,123}.

Dataset	Precision	Recall	${\mathrm{mAP}}_{50}$	${\mathrm{mAP}}_{50:95}$
VisDrone	0.5101±0.0044	0.4094±0.00425	0.4054±0.0032	0.2308±0.0023
UAVDT	0.8275±0.0099	0.8242±0.0118	0.8591±0.0036	0.5401±0.0012
VEDAI	0.7876±0.0441	0.6258±0.0725	0.6984±0.0603	0.4253±0.0267

**Table 9:** Matched three-seed reproducibility analysis on VisDrone. Values are reported as mean ± sample standard deviation across seeds {5,42,123}.

Configuration	Precision	Recall	${\mathrm{mAP}}_{50}$	${\mathrm{mAP}}_{50:95}$
YOLOv8n	0.5010±0.0057	0.4120±0.0018	0.4082±0.0030	0.2311±0.0012
RIF-YOLO-N w/o RGFT	0.4860±0.0111	0.4229±0.0015	0.3980±0.0032	0.2364±0.0021
RIF-YOLO-N + RGFT	0.5040±0.0091	0.4140±0.0034	0.4110±0.0006	0.2410±0.0005

**Table 10:** Latency is measured at 1024×1024 using the same controlled batch-size-one runtime protocol for both models.

Model	Params	GFLOPs	Inf. Time	Main Design
YOLOv8n	3.008M	8.1	18.272±6.489 ms	Standard anchor-free P3/P4/P5 detector
RIF-YOLO-N	3.061M	8.8	19.035±5.322 ms	Identity-safe P3 enhancement + RGFT
Δ	+0.053M	+0.7	+0.763 ms	–

**Table 11:** Controlled resolution-sensitivity comparison on the VisDrone validation set.

Model	Resolution	P	R	${\mathrm{mAP}}_{50}$	${\mathrm{mAP}}_{50:95}$
YOLOv8n	640×640	0.442	0.350	0.326	0.184
YOLOv8n	768×768	0.479	0.379	0.362	0.207
YOLOv8n	832×832	0.490	0.387	0.374	0.216
YOLOv8n	960×960	0.497	0.408	0.389	0.226
YOLOv8n	1024×1024	0.507	0.411	0.401	0.235
YOLOv8n + RGFT	1024×1024	0.511	0.415	0.405	0.238
RIF-YOLO-N w/o RGFT	640×640	0.437	0.356	0.330	0.186
RIF-YOLO-N w/o RGFT	768×768	0.481	0.381	0.363	0.207
RIF-YOLO-N w/o RGFT	832×832	0.494	0.388	0.377	0.215
RIF-YOLO-N w/o RGFT	960×960	0.491	0.409	0.388	0.223
RIF-YOLO-N w/o RGFT	1024×1024	0.496	0.422	0.402	0.232
RIF-YOLO-N + RGFT	640×640	0.464	0.349	0.328	0.185
RIF-YOLO-N + RGFT	768×768	0.485	0.382	0.367	0.212
RIF-YOLO-N + RGFT	832×832	0.505	0.391	0.381	0.221
RIF-YOLO-N + RGFT	960×960	0.517	0.407	0.401	0.234
RIF-YOLO-N + RGFT	1024×1024	0.520	0.424	0.415	0.243

**Table 12:** Controlled comparison on the UAVDT validation set.

Model	Input Resolution	P	R	${\mathrm{mAP}}_{50}$	${\mathrm{mAP}}_{50:95}$
YOLOv8n	640×640	0.871	0.791	0.853	0.523
YOLOv8n	832×832	0.850	0.811	0.844	0.527
YOLOv8n	1024×1024	0.845	0.814	0.833	0.519
RIF-YOLO-N w/o RGFT	640×640	0.844	0.775	0.850	0.500
RIF-YOLO-N w/o RGFT	832×832	0.840	0.810	0.826	0.492
RIF-YOLO-N w/o RGFT	1024×1024	0.812	0.820	0.814	0.471
RIF-YOLO-N + RGFT	640×640	0.882	0.814	0.870	0.513
RIF-YOLO-N + RGFT	832×832	0.848	0.841	0.865	0.534
RIF-YOLO-N + RGFT	1024×1024	0.832	0.838	0.862	0.543

**Table 13:** Controlled VEDAI comparison at 1024×1024, showing the mAP gains of RIF-YOLO-N + RGFT over YOLOv8n.

Model	Input Resolution	P	R	${\mathrm{mAP}}_{50}$	${\mathrm{mAP}}_{50:95}$
YOLOv8n	640×640	0.668	0.499	0.558	0.356
YOLOv8n	832×832	0.582	0.525	0.573	0.355
YOLOv8n	1024×1024	0.387	0.479	0.409	0.252
RIF-YOLO-N w/o RGFT	640×640	0.628	0.684	0.711	0.379
RIF-YOLO-N w/o RGFT	832×832	0.752	0.484	0.633	0.359
RIF-YOLO-N w/o RGFT	1024×1024	0.640	0.391	0.414	0.232
RIF-YOLO-N + RGFT	640×640	0.394	0.370	0.413	0.201
RIF-YOLO-N + RGFT	832×832	0.788	0.558	0.716	0.427
RIF-YOLO-N + RGFT	1024×1024	0.799	0.586	0.669	0.419

**Table 14:** Structural comparison of representative aerial small-object detectors. The notation P2–P5 indicates the feature levels or prediction scales emphasized by each method when reported in the corresponding source.

Method	Detector Family	Main Feature Levels/Strategy	P2 Use	Dataset/Split	Main Design Characteristic
LEAF-YOLO-N [31]	YOLOv7-tiny style	P2/P3/P4/P5	Yes	VisDrone2019-DET-val	Lightweight edge-oriented model using multi-scale high-resolution detection for small aerial objects.
LEAF-YOLO [31]	YOLOv7-tiny style	P2/P3/P4/P5	Yes	VisDrone2019-DET-val	Larger LEAF variant with stronger multi-scale feature aggregation and higher accuracy.
YOLC [32]	CenterNet-based	Cluster-region zooming + CenterNet head	Not YOLO-scale based	VisDrone2019-DET-val	Detects object clusters, zooms into dense regions, and refines tiny-object localization.
TPH-YOLOv5 [33]	YOLOv5-based	P3/P4/P5 + extra small-object head	Yes	VisDrone2021	Adds a transformer prediction head and attention modules for drone-captured dense scenes.
EdgeYOLO [34]	YOLO-based	Anchor-free multi-scale YOLO head	Not explicitly fixed in this table	VisDrone2019-DET	Edge-real-time anchor-free detector with lightweight decoupled head and small-object-oriented loss design.
RIF-YOLO-N	YOLOv8n-based	P3/P4/P5 with enhanced P3	No	VisDrone validation	Identity-safe same-scale P3 enhancement with resolution-guided fine-tuning; no additional detection head.

**Table 15:** Reported complexity and inference information of representative aerial object detectors. The values are taken from the corresponding studies and are provided for contextual comparison only, as the input resolutions, hardware platforms, and timing protocols do not match.

Method	Params	GFLOPs	Reported Inference/Speed
LEAF-YOLO-N [31]	1.20M	5.6	16.2 ms
LEAF-YOLO [31]	4.28M	20.9	21.7 ms
YOLC [32]	NA	NA	441 ms
TPH-YOLOv5 [33]	NA	315.4	7.36 FPS
EdgeYOLO-T [34]	5.50M	27.24	29.93 ms
RIF-YOLO-N (ours)	3.061M	8.8	19.035 ms

**Table 16:** Literature-reported detection performance of representative object detectors across VisDrone, UAVDT, and VEDAI. AP denotes ${\mathrm{mAP}}_{50:95}$ and ${\mathrm{AP}}_{50}$ denotes mAP at IoU 0.5. NA indicates that the corresponding result was not available in the cited source. The results are not protocol matched because the methods use different input resolutions, dataset splits, training schedules, augmentation strategies, and evaluation settings; therefore, the values are provided only for contextual positioning and no direct superiority claim is inferred from this table.

Method	VisDrone			UAVDT			VEDAI		
	Input	AP (%)	${\mathrm{AP}}_{50}$ (%)	Input	AP (%)	${\mathrm{AP}}_{50}$ (%)	Input	AP (%)	${\mathrm{AP}}_{50}$ (%)
LEAF-YOLO-N [31]	640	21.9	39.7	NA	NA	NA	NA	NA	NA
LEAF-YOLO [31]	640	28.2	48.3	NA	NA	NA	NA	NA	NA
YOLC [32]	1024	31.8	55.0	1024	19.3	30.9	NA	NA	NA
TPH-YOLOv5 [33]	1536	34.0	53.2	1024	26.9	41.3	NA	NA	NA
EdgeYOLO-T [34]	640	21.8	38.5	NA	NA	NA	NA	NA	NA
RIF-YOLO-N + RGFT (ours)	1024	24.3	41.5	1024	54.3	86.2	1024	41.9	66.9

**Table 17:** Computational-efficiency context combining the controlled YOLOv8n–RIF-YOLO-N benchmark with literature-reported values for representative tiny- and small-object detectors. External values are reproduced from the corresponding studies and are not protocol-matched because input resolution, hardware platform, batch size, and runtime measurement procedures may differ. Only YOLOv8n and RIF-YOLO-N are measured under the same controlled protocol and are therefore used for direct latency and throughput comparison.

Method	Input	Params	GFLOPs	Latency (ms)	${\mathrm{FPS}}_{b=1}$
LEAF-YOLO-N [31]	640	1.20M	5.6	16.2	56
LEAF-YOLO [31]	640	4.28M	20.9	21	32
EdgeYOLO-T [34]	640	5.50M	27.24	–	27
YOLC [32]	1024	–	151.0	441	–
TPH-YOLOv5 [33]	1536	–	315.4	–	7.36
YOLOv8n	1024	3.008M	8.1	18.272±6.489	54.73
RIF-YOLO-N	1024	3.061M	8.8	19.035±5.322	52.54

**Table 18:** Object-size distribution in the VisDrone validation set.

Object Size	Area Range	Instances	Ratio
Tiny	A<0.001	25,967	67.00%
Small	0.001≤A<0.01	11,894	30.69%
Medium	0.01≤A<0.05	871	2.25%
Large	A≥0.05	27	0.07%

**Table 19:** Size-stratified ${\mathrm{AP}}_{50}$ comparison on the VisDrone validation set.

Object Size	Instances	YOLOv8n	RIF-YOLO-N	Gain
Tiny	25,967	0.1298	0.1346	+0.0048
Small	11,894	0.3771	0.3852	+0.0081

**Table 20:** Class-wise AP comparison between YOLOv8n and RIF-YOLO-N on the VisDrone validation set at 1024×1024.

Class	Instances	YOLOv8n ${\mathrm{AP}}_{50}$	RIF-YOLO-N ${\mathrm{AP}}_{50}$	${\Delta \mathrm{AP}}_{50}$	YOLOv8n ${\mathrm{AP}}_{50:95}$	RIF-YOLO-N ${\mathrm{AP}}_{50:95}$	${\Delta \mathrm{AP}}_{50:95}$
Pedestrian	8844	0.474	0.493	+0.019	0.212	0.223	+0.011
People	5125	0.334	0.361	+0.027	0.121	0.134	+0.013
Bicycle	1287	0.124	0.155	+0.031	0.051	0.066	+0.014
Car	14,064	0.823	0.829	+0.006	0.568	0.575	+0.007
Van	1975	0.454	0.486	+0.032	0.318	0.342	+0.025
Truck	750	0.342	0.372	+0.031	0.222	0.248	+0.026
Tricycle	1045	0.277	0.265	−0.012	0.155	0.149	−0.006
Awning-tricycle	532	0.156	0.137	−0.019	0.101	0.086	−0.015
Bus	251	0.552	0.559	+0.008	0.395	0.396	+0.001
Motor	4886	0.477	0.492	+0.015	0.203	0.216	+0.012

**Table 21:** Detection diagnostic outputs used for qualitative and error-pattern analysis.

Diagnostic Output	Purpose	Relevance to This Study
Precision–recall curve	Measures the relation between precision and recall across confidence thresholds.	Indicates whether the final detector preserves detection reliability while improving object recovery.
F1-confidence curve	Shows the confidence region where precision and recall are balanced.	Helps identify whether the model maintains stable confidence behavior under dense tiny-object conditions.
Normalized confusion matrix	Reports class-level prediction patterns and background confusion.	Explains remaining errors for visually similar or low-frequency classes such as tricycle and awning-tricycle.
Qualitative prediction samples	Shows predicted bounding boxes on validation images.	Verifies whether numerical improvements correspond to visible recovery of small and dense objects.

**Table 22:** Ablation study of RIF-YOLO-N on the VisDrone validation set at 1024×1024. Each architecture-level variant removes only the component identified in its name while keeping the remaining RIF components unchanged.

Variant	Params	GFLOPs	P	R	${\mathrm{mAP}}_{50}$	${\mathrm{mAP}}_{50:95}$
YOLOv8n baseline	3.008M	8.1	0.507	0.411	0.401	0.235
Complete RIF w/o RGFT	3.061M	8.8	0.496	0.422	0.402	0.232
RIF w/o projection	3.058M	8.8	0.503	0.411	0.396	0.228
RIF w/o gate	3.026M	8.4	0.496	0.409	0.394	0.228
RIF w/o refinement	3.029M	8.4	0.487	0.416	0.391	0.226
RIF w/o residual scale	3.034M	8.5	0.485	0.416	0.394	0.227
RIF + RGFT (final)	3.061M	8.8	0.520	0.424	0.415	0.243

**Table 23:** Design-space comparison of the architecture variants considered during RIF-YOLO-N development.

Architecture Variant	Detection Scales	P2 Use	P5 Retained	Enhancement Strategy	Design Purpose
RIF-YOLO-N final	P3/P4/P5	No	Yes	Same-scale identity-safe P3 enhancement	Preserve the lightweight YOLOv8n hierarchy while improving the P3 representation.
P2/P3/P4 without P5	P2/P3/P4	Yes	No	High-resolution detection without the P5 semantic branch	Test whether adding high-resolution detection while removing high-level semantics benefits tiny objects.
Heavy LEAF-style V3	P2/P3/P4/P5	Yes	Yes	High-resolution context and stronger feature aggregation	Test whether heavier high-resolution processing improves AP enough to justify the added complexity.
V3-Efficient	P3/P4/P5	Internal P2 guidance	Yes	Lightweight P2-guided enhancement	Test whether P2 guidance can improve accuracy without a large computational increase.

**Table 24:** Architecture selection results on the VisDrone validation set at 1024×1024.

Architecture Variant	Params	GFLOPs	P	R	${\mathrm{mAP}}_{50}$	${\mathrm{mAP}}_{50:95}$	Selection Outcome
RIF-YOLO-N final	3.061M	8.8	0.520	0.424	0.415	0.243	Selected because it provides the best accuracy–complexity balance.
P2/P3/P4 without P5	2.099M	12.3	0.463	0.386	0.360	0.209	Not selected because removing P5 reduced semantic context and AP.
Heavy LEAF-style V3	5.159M	38.6	0.510	0.410	0.404	0.236	Not selected because stronger high-resolution and context operations increased complexity but did not exceed the final RIF-YOLO-N result.
V3-Efficient	3.064M	8.4	0.472	0.408	0.383	0.221	Not selected because lightweight P2-guided enhancement produced lower AP.

**Table 25:** Relative difference of alternative architectures compared with the final RIF-YOLO-N. Negative AP values indicate performance loss relative to the final model.

Architecture Variant	ΔParams	ΔGFLOPs	ΔP	ΔR	${\Delta \mathrm{mAP}}_{50}$	${\Delta \mathrm{mAP}}_{50:95}$
P2/P3/P4 without P5	−0.962M	+3.5	−0.057	−0.038	−0.055	−0.034
Heavy LEAF-style V3	+2.098M	+29.8	−0.010	−0.014	−0.011	−0.007
V3-Efficient	+0.003M	−0.4	−0.048	−0.016	−0.032	−0.022

## Data Availability

The code is available at [35]. The experiments use the VisDrone [28], UAVDT [29], and VEDAI [30] datasets. These datasets are not redistributed with the source code and should be obtained from their official sources.
